# Bile Acid Conjugation on Solid Nanoparticles Enhances ASBT‐Mediated Endocytosis and Chylomicron Pathway but Weakens the Transcytosis by Inducing Transport Flow in a Cellular Negative Feedback Loop

**DOI:** 10.1002/advs.202201414

**Published:** 2022-06-02

**Authors:** Feiyang Deng, Kyoung Sub Kim, Jiyoung Moon, You Han Bae

**Affiliations:** ^1^ Department of Pharmaceutics and Pharmaceutical Chemistry College of Pharmacy University of Utah 30 S 2000 E Salt Lake City UT 84112 USA

**Keywords:** apical sodium dependent bile acid transporter (ASBT), bile acid‐modified nanoparticles, chylomicron pathway, transcytosis, transport feedback

## Abstract

Bile acid‐modified nanoparticles provide a convenient strategy to improve oral bioavailability of poorly permeable drugs by exploiting specific interactions with bile acid transporters. However, the underlying mechanisms are unknown, especially considering the different absorption sites of free bile acids (ileum) and digested fat molecules from bile acid‐emulsified fat droplets (duodenum). Here, glycocholic acid (GCA)‐conjugated polystyrene nanoparticles (GCPNs) are synthesized and their transport in Caco‐2 cell models is studied. GCA conjugation enhances the uptake by interactions with apical sodium‐dependent bile acid transporter (ASBT). A new pathway correlated with both ASBT and chylomicron pathways is identified. Meanwhile, the higher uptake of GCPNs does not lead to higher transcytosis to the same degree compared with unmodified nanoparticles (CPNs). The pharmacological and genomics study confirm that GCA conjugation changes the endocytosis mechanisms and downregulates the cellular response to the transport at gene levels, which works as a negative feedback loop and explains the higher cellular retention of GCPNs. These findings offer a solid foundation in the bile acid‐based nanomedicine design, with utilizing advantages of the ASBT‐mediated uptake, as well as inspiration to take comprehensive consideration of the cellular response with more developed technologies.

## Graphical abstract

Glycocholic acid‐modification endows specific transcytosis pathways to carboxylated polystyrene nanoparticle (CPN) in Caco‐2 cells. The modified nanoparticle (GCPN) is internalized by apical sodium dependent bile acid transporter (ASBT) via unique endocytosis mechanisms and shared the pathway with chylomicron. The caco‐2 cells slow GCPN transcytosis rate by weakening the transport‐relevant pathways.

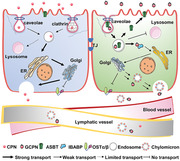

## 1 Introduction

Oral administration is desirable in clinical therapy due to its noninvasive and convenient nature, leading to higher patient compliance and lower overall costs.^[^ ^1^, ^2^, ^3^ ^]^ A vast number of drugs experience low oral bioavailability due to nonideal physicochemical properties, such as poor solubility, low permeability, and enzymatic digestion.^[^ ^4^ ^]^ With nanosuspension technologies, the oral absorption of poorly soluble drugs is significantly enhanced by improving their dissolution rates.^[^ ^5^ ^]^ For drugs with low permeability, however, additional strategies must be employed.^[^ ^6^ ^]^ One attractive method to improve permeability currently involves targeting intestinal receptor/transporter systems. For example, vitamin B_12_‐modification has been reported to increase oral absorption by targeting the receptor cubilin.^[^ ^7^, ^8^ ^]^ Despite this, the low absorption capacity of B_12_ (1–2 µg per day in humans) limited its application owing to the difficulty in meeting the therapeutic windows of drugs.^[^ ^9^ ^]^ Therefore, there is an urgency to develop a preferable strategy to improve oral bioavailability.

Bile acids and their transporters have been attracting the interest of researchers in oral administration in recent years for their specific biological processes. Bile acids are amphiphilic molecules synthesized and secreted by the liver and stored in the gallbladder.^[^ ^10^ ^]^ At meals, bile acids transport to the duodenum to assist the emulsification and digestion of dietary fats. In the ileum, the bile acids are reabsorbed by enterocytes to portal circulation and recycled back to the liver. In this whole biological process called “enterohepatic recycling,” only ≈5% of the large bile acid pool (3 g) is excreted.^[^ ^11^, ^12^ ^]^ The high capacity and absorption rate of bile acids in the small intestine (the body recycles the pool approximately six times a day) demonstrate their potential in the application of oral delivery.

Apical sodium‐dependent bile acid transporter (ASBT) is primarily expressed in the apical side of the ileal enterocytes and is regarded as the most critical molecular transporter for high bile acid reabsorption efficacy.^[^ ^13^ ^]^ Therefore, many studies have focused on the ASBT‐targeting strategy, utilizing the ASBT‐bile acid interactions to improve the oral drug delivery of poorly absorbed drugs. For instance, Khatun et al. developed taurocholic acid‐linked heparin‐docetaxel nanoparticles as an oral ASBT‐targeting system, which exhibited a 63% decrease in tumor volume compared with docetaxel after oral administration in SKH1 mice.^[^ ^14^ ^]^ Our group grafted glycocholic acid to chondroitin sulfate (CS) and coated it on exendin‐4‐loaded cationic liposomes, which showed significantly higher oral bioavailability (≈20%) compared with the CS‐coated liposomes.^[^ ^15^ ^]^ Such studies revealed the immense potential of ASBT‐mediated oral drug delivery.

Contrary to the flourishing of ASBT‐mediated oral administration studies, the underlying transport pathways of the bile acid‐modified formulations remain largely unknown. Free bile acids have been proven to interact with ileal bile acid binding protein (IBABP) after endocytosis into the enterocytes.^[^ ^16^ ^]^ Subsequently, bile acids transport across the basolateral membrane with the assistance of organic solute transporter (OST*α*/*β*).^[^ ^17^ ^]^ However, it is not fully elucidated whether nanoparticle (NP) formulations will alter this route. Free glycocholic acid (GCA) could be pumped in by ASBT with co‐transport of two Na^+^ ions, while it is unlikely that GCA‐conjugated polystyrene nanoparticles (GCPNs) with the size of around 100 nm could permeate through the narrow cavity of ASBT (6 Å × 12 Å × 14 Å).^[^ ^18^ ^]^ In addition, dietary fats emulsified with bile acids are absorbed in the duodenum and transported to the lymphatic system as chylomicron, where ASBT expression is relatively low.^[^ ^19^ ^]^ In 2014, Al‐Hilal and co‐workers conjugated the tetrameric deoxycholic acids to low molecular weight heparin (LHe‐*tetraD*) and investigated its transport in SK‐BR‐3 cells (a breast cancer line with high ASBT expression).^[^ ^20^ ^]^ It was found that nanosized LHe‐*tetraD* demonstrated high ASBT colocalization, indicating their transport is mediated by ASBT. Our group further studied the oral administration behavior of GCPNs, and we found they accumulated in the lymphatic system in rats.^[^ ^21^ ^]^ These studies demonstrate the complexity of bile acid‐conjugated NP absorption.

To clarify the oral transport mechanisms of bile acid‐modified NPs, GCPN studies were executed in ASBT‐expressing Caco‐2 cells, a standard model in oral transport study. The endocytosis, intracellular transport, transcytosis, and paracellular transport of the GCPN were investigated and compared with those of CPN at protein and gene levels using receptor inhibition tests, gene knockdown analysis, and confocal fluorescence microscopy colocalization studies. GCPN participation in the chylomicron pathway was also studied with lipoprotein isolation. In‐depth genomic studies were used to investigate more profound levels of transport regulation.

## 2 Results and Discussion

### 2.1 Preparation and Characterization of GCPN

Commercial CPN with a defined size of 100 nm, labeled with a red fluorescence, was used as an inert probe to study the transport pathways due to their stability under biological conditions. The preparation and characterization of GCPN have been described in our previous studies.^[^ ^21^ ^]^ The presence of the corresponding methyl groups of GCA in the final product in NMR spectrum confirmed the successful conjugation of GCA onto the CPN surface. With GCA conjugation, GCPN had a slightly larger particle size and an increased (less negative) Zeta potential due to the consumption of carboxyl groups. The transmission electron microscope (TEM) images showed that both nanoparticles were of regular spherical shape (Figure S1, Supporting Information).

### 2.2 Endocytosis Pathway of CPN and GCPN

Our previous study confirmed the higher uptake efficiency of GCPN than that of CPN in SK‐BR‐3 cells, which we attributed to the ASBT‐mediated endocytosis.^[^ ^21^ ^]^ Although SK‐BR‐3 cells have been reported to display epithelial morphology,^[^ ^22^ ^]^ there is no evidence that this cell line could form intact and stable polar cell monolayers with differentiated apical and basolateral membranes, limiting its application in mimicking enterocytes in oral administration studies, especially in the transcytosis exploration. Therefore, in this study, Caco‐2 cells were selected due to their ability to express several morphological and functional characteristics of the mature enterocyte.^[^ ^23^ ^]^ For oral bile acid‐based drug delivery studies, Caco‐2 cell line has been widely used as a standard model for years.^[^ ^24^, ^25^, ^26^ ^]^

**Figure 1:**
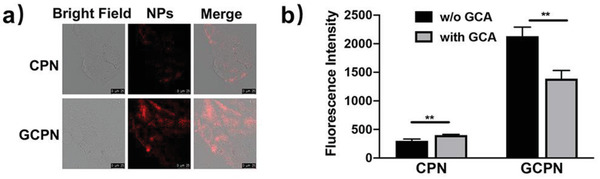
a) CLSM analysis of cellular uptake of CPN and GCPN in Caco‐2 cells in 0.5 h. GCPN demonstrated much higher uptake efficiency. b) Competitive effect of free GCA on the uptake of CPN and GCPN. Free GCA inhibited the uptake of GCPN by ≈40%, while slightly inducing the CPN uptake. **, *p* < 0.01.

The uptake behaviors of GCPN and CPN are shown in **Figure** **1**. GCPN demonstrated significantly higher uptake (approximately six‐fold) than CPN in 0.5 h in Caco‐2 cells, consistent with our previous study in SK‐BR‐3 cells.^[^ ^21^ ^]^ To investigate the role of ASBT in endocytosis, free GCA was added to compete with GCPNs for ASBT binding sites. The resulting uptake of GCPN was remarkably decreased by the competition of GCA, while no inhibition was found on CPN uptake, suggesting the dependence on the interaction between GCPN and ASBT for endocytosis. According to our previous study, each CPN was conjugated with 24 GCA molecules,^[^ ^21^ ^]^ and the particle concentration was 1.9 × 10^11^ mL^−1^ at 100 µg mL^−1^. Thus, the molar ratio between the free and conjugated GCA was ≈270 000:1. However, the disparity in concentration only demonstrated a 40% inhibition effect. This implies there is difference between the uptake mechanisms of GCPN and free GCA, although both involve ASBT. GCPNs were expected to be internalized via an endocytic pathway, with more complicated regulation and the assistance of cell components to open the binding sites and interact with ASBT.

**Figure 2:**
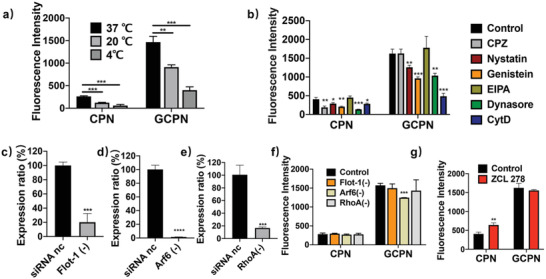
Endocytosis mechanisms of CPN and GCPN in Caco‐2 cells. Effects of a) temperature and b) pharmacological inhibitors on the endocytosis of CPN and GCPN in Caco‐2 cells. Endocytosis of both NPs was reduced at lower temperatures. Nystatin, genistein, dynasore, and CytD inhibited the uptake of both CPN and GCPN, indicating the participation of caveolae, dynamin, and actin filaments in the endocytosis. CPZ inhibited CPN uptake but not GCPN, suggesting only CPNs were internalized by clathrin‐mediated pathway. c–e) RT‐PCR analysis of expression of c) Flot‐1, d) Arf6, and e) RhoA after knocking down by siRNA. All of the mRNA expression decreased by over 80% compared with nonspecific siRNA (siRNA nc). f) Effect of Flot‐1, Arf6, and RhoA on the endocytosis of CPN and GCPN. Only knocking down of Arf6 showed inhibition of GCPN uptake. g) Effect of Cdc42 on the endocytosis of CPN and GCPN. ZCL278 induced the uptake of CPN but did not influence GCPN. *, *p* < 0.05; **, *p* < 0.01; ***, *p* < 0.001; ****, *p* < 0.0001.

To elucidate the particular endocytosis pathways, the effects of temperature and pharmacological inhibitors were tested. As shown in **Figure** **2**a, both CPN and GCPN showed temperature‐dependent uptake in Caco‐2 cells, with decreasing endocytosis at 20 °C and stronger uptake inhibition at 4 °C. It is known that endocytosis is an active process, which is energy‐dependent at 37 °C and can be inhibited by low temperature (4 °C).^[^ ^27^ ^]^ At around 20 °C, many abnormal changes could be induced, including an irregularly extended network of labyrinthine channels, coated pits and vesicles, tubular elements, and alpha vacuoles.^[^ ^28^ ^]^ Besides, at the inflection point (20–25 °C), cell membrane morphology is also changed in microdomain separation, liquid phase transition, and protein conformational changes.^[^ ^29^ ^]^ These results indicate that uptake of both CPN and GCPN depends on an energy‐dependent endocytic way and relies on the normal cell membrane morphology.

The effect of various pharmacological inhibitors on cellular viability was assessed using the 3‐(4,5‐dimethylthiazol‐2‐yl)‐2,5‐diphenyltetrazolium bromide (MTT) assay (Figure S2, Supporting Information). All the inhibitors showed little cytotoxicity in Caco‐2 cells at concentrations used in this study, suggesting they did not interfere with any critical biological functions besides the corresponding endocytosis process. Figure 2b displays the effect of the inhibitors on CPN and GCPN internalization in Caco‐2 cells. The mechanisms of the inhibitors are summarized in Table S1 in the Supporting Information. Nystatin, genistein, dynasore, and CytD inhibited the endocytosis of both CPN and GCPN at varying degrees, indicating that both CPN and GCPN are internalized via the caveolae‐mediated pathway, driven by actin filaments and with the assistance of dynamin for budding of vesicles in Caco‐2 cells. On the contrary, 5‐(*N*‐ethyl‐*N*‐isopropyl) amiloride (EIPA) did not show an inhibition effect in CPN or GCPN uptake. Thus, macropinocytosis was not involved in the uptake of CPN or GCPN. The most apparent difference between CPN and GCPN uptake was the effect of chlorpromazine (CPZ), the inhibitor of the clathrin‐mediated pathway. Clathrin‐mediated endocytosis is one of the most common vesicular trafficking processes for cargos transported from cell surface to interior space.^[^ ^30^ ^]^ As shown in Figure 2b, endocytosis of CPN was significantly hindered by CPZ treatment, while no effect was observed on GCPN in Caco‐2 cells, indicating GCA‐conjugation altered the NPs transport route away from the clathrin‐mediated pathway.

It is reasonable that the uptake of CPN and GCPN was inhibited by dynamin because both clathrin‐ and caveolae‐mediated endocytosis are dependent on its activity.^[^ ^31^ ^]^ However, some other uptake mechanisms, called “clathrin/caveolae‐independent endocytosis,” may (Flot‐1, RhoA) or may not (cdc42, Arf6) relevant to the dynamin.^[^ ^31^, ^32^ ^]^ A specific description of these routes is listed in Table S2 in the Supporting Information. Although not as typical as clathrin/caveolae‐mediated endocytosis, they might also be involved in the uptake of CPN/GCPN. To clarify this issue, Flot‐1, Arf6, and RhoA were knocked down by siRNA transfection, and their expression was tested by quantitative polymerase chain reaction (qPCR; Figure 2c–e). For cdc42, ZCL278 was used to inhibit its effect.^[^ ^33^ ^]^ As shown in Figure 2d,e, the uptake of GCPN was partially blocked by Arf6 silencing, and the CPN internalization was enhanced by cdc42 inhibition. This result suggests that CPN and GCPN transports were diversified in the clathrin/caveolae‐independent endocytosis process. For GCPN, only Arf6 participated in the internalization, while a more complex mechanism was involved in CPN uptake. The inhibition of cdc42 led to a higher uptake rate, which might be due to the activation of other pathways. Therefore, cdc42 at least responded to the uptake of CPN and played some roles in this process.

### 2.3 Intracellular Pathways of CPN and GCPN in Caco‐2 Cells

**Figure 3:**
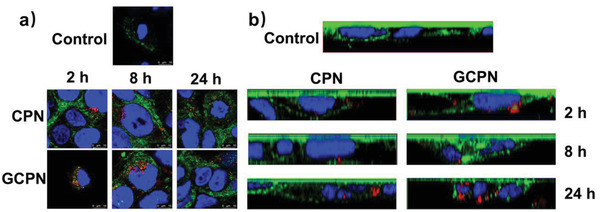
CLSM analysis of ASBT distribution and colocalization with CPN and GCPN at 2, 8, and 24 h in Caco‐2 cells: a) *X*–*Y* images; b) *X*–*Z* images. CPN did not colocalize with ASBT and showed little influence on its distribution. GCPN colocalized with ASBT at 2 h and gradually separated from it in 8 h. ASBT was internalized into the cytoplasm in 8 h and recycled back to the cell membrane in 24 h.

In our previous study, we observed the recycling of ASBT between the cell membrane and intracellular space during the endocytosis and intracellular transport of GCPN in SK‐BR‐3 cells.^[^ ^21^ ^]^ The result in the endocytosis study confirmed that uptake of GCPN was dependent on the activity of ASBT, but CPN was not. To further explore how ASBT worked in Caco‐2 cells, the ASBT distribution was observed by confocal laser scanning microscope (CLSM). As shown in **Figure** **3**, ASBT was mainly located on the cell membrane without NPs treatment. Incubation with CPN for different periods (2, 8, and 24 h) did not impact the distribution of ASBT, and no colocalization between ASBT and CPN was observed. By contrast, GCPN was merged with ASBT after incubation for 2 h near the cell membrane. At 8 h, ASBT was largely internalized into the cells, disassociated with GCPN, and then slowly recycled to the membrane in 24 h. The GCPN‐ASBT interaction–disassociation and ASBT‐recycling modes in Caco‐2 cells were consistent with the previous studies in SK‐BR‐3 cells,^[^ ^20^, ^21^ ^]^ indicating that the ASBT‐mediated process required the accompaniment of ASBT with the cargos in various models.

**Figure 4:**
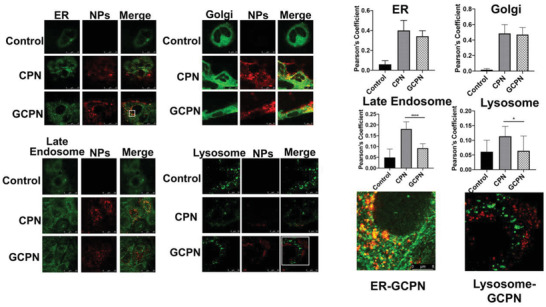
Colocalization images and Pearson's coefficient of CPN and GCPN (red) with ER, Golgi apparatus, late endosomes, and lysosomes (green) in Caco‐2 cells. The white square parts were shown in higher magnification. Both CPN and GCPN showed colocalization with ER and Golgi (higher Pearson's coefficient) but little with Rab 7 (late endosomes) and lysosomes. Compared with CPN, GCPN demonstrated less involvement in the maturation process.

Generally, lysosomal degradation and exocytosis are the two primary fates of internalized NPs. To study the intracellular pathway, the endoplasmic reticulum (ER), Golgi apparatus, late endosomes, and lysosomes were stained and the colocalization between these organelles and NPs was studied. ER and Golgi apparatus are crucial compartments for endosomal recognition and secretary process.^[^ ^34^, ^35^ ^]^ In the cellular acidification process, late endosomes (marker: rab7) mature from early endosomes before fusing with lysosomes, the cellular degradation center, which plays a role in maintaining homeostasis and a barrier to transmembrane delivery of drugs.^[^ ^36^ ^]^ As shown in **Figure** **4**, both NPs showed significant colocalization with ER and Golgi apparatus, with Pearson's coefficient much higher than the background. On the contrary, the merging between NPs and late endosomes or lysosomes was limited. With a much lower Pearson's coefficient, this result indicates that neither of the two NPs was confined in the lysosomal pathway, despite the statistical difference shown between the two NPs.

**Figure 5:**
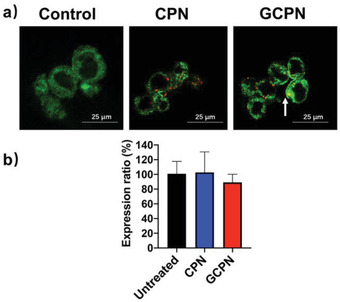
a) Colocalization of CPN/GCPN (red) with IBABP (green) in Caco‐2 cells. The white arrow indicates colocalization. b) RT‐PCR analysis of IBABP expression after CPN and GCPN treatment.

After disassociation with ASBT, the interaction between GCPN and IBABP was investigated. In the ileum, IBABP binding is essential for the transport of free bile acids to the basolateral membrane.^[^ ^13^ ^]^ As **Figure** **5**a shows, CPN was dispersed in the cytoplasm with rare colocalization with IBABP. In contrast, significant overlap was observed between GCPN and IBABP, illustrating that transport of GCPN was dependent on the interaction with IBABP, as is the case with free bile acids do. However, GCPN did not affect the expression of IBABP in Caco‐2 cells (Figure 5b). It is known that IBABP expression is mainly regulated by the farnesoid X receptor (FXR), a nuclear receptor activated by bile acids binding.^[^ ^37^, ^38^ ^]^ The FXR‐bile acid complexes are then translocated into the nuclei and bind to the specific DNA sequence.^[^ ^39^ ^]^ In this way, the transcription of IBABP gene can be regulated. With free bile acids treatment, the IBABP expression is expected to be elevated.^[^ ^16^ ^]^ However, unlike FXR‐bile acid complexes, the FXR‐GCPN complexes, if formed, could hardly enter the nuclei for the transactivation effect. This might account for why GCPN did not upregulate IBABP expression.

### 2.4 Transmembrane Study of CPN and GCPN across the Caco‐2 Monolayer

**Figure 6:**
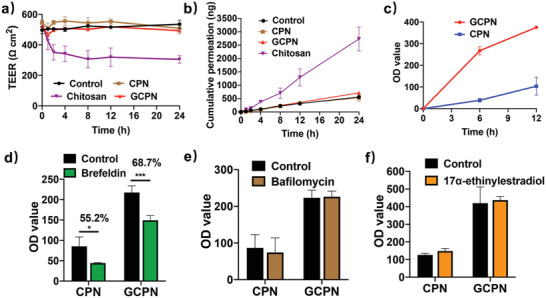
a) TEER of Caco‐2 monolayers with the treatment of chitosan, CPN, and GCPN. No significant TEER change occurred with the treatment of CPN and GCPN in 24 h. b) Cumulative permeation of Na‐Flu across the Caco‐2 monolayers with the treatment of chitosan, CPN, and GCPN. CPN and GCPN did not influence Na‐Flu permeation. c) Quantitative analysis of transcytosis of CPN and GCPN across the Caco‐2 monolayers. d–f) Effect of brefeldin, bafilomycin, and 17*α*‐ethinylestradiol on the transcytosis of CPN and GCPN across the Caco‐2 monolayers. Brefeldin reduced the transcytosis of CPN and GCPN, while bafilomycin and 17*α*‐ethinylestradiol did not disturb their transport. *, *p* < 0.05; ***, *p* < 0.001.

Under steady‐state conditions, the pore size of the tight junction is only ≈8 Å, limiting the transport of NPs.^[^ ^36^ ^]^ Therefore, monitoring the tight junction's integrity could tell whether the CPN and GCPN are transported through the paracellular pathway (**Figure** **6**a,b). Chitosan is known to open the tight junction and lead to transepithelial electrical resistance (TEER) reduction by translocation of tight junction proteins from the membrane to the cytoskeleton.^[^ ^40^ ^]^ Compared with the chitosan group, neither of the two NPs showed TEER reduction or Na‐Flu permeation in 12 h. These results indicated that CPN and GCPN did not open the tight junction, and the two NPs were transported through transcytosis rather than paracellular pathway.

Figure 6c displays the transcytosis behaviors of CPN and GCPN in 12 h. GCPN showed an approximately three‐fold transcytosis ratio to CPN in 12 h. This was consistent with the 3D model of the NPs distribution in the monolayer (Videos S1–S3, Supporting Information). GCPN had more adhesion on the apical side, which might be due to the affinity to ASBT. The intracellular and basolateral GCPN intensity was higher than that of CPN, suggesting higher transmembrane permeability. Various inhibitors were used to explore the specific transcytosis mechanism (Table S4, Supporting Information). Brefeldin is an inhibitor blocking the trafficking from ER to Golgi by triggering fragmentation of the Golgi apparatus.^[^ ^41^ ^]^ Bafilomycin specifically inhibits lysosomal degradation by blocking the vacuolar type H^+^‐ATPase.^[^ ^42^ ^]^ Within the 12 h time point, the inhibitors did not disrupt the integrity of the monolayer (Figure S3, Supporting Information). As Figure 6d,e shows, brefeldin treatment caused a significant reduction of transcytosis of CPN and GCPN, while bafilomycin affected neither of the two. This result confirmed that both CPN and GCPN transported through the ER‐Golgi pathway but did not participate in the intracellular acidification process, which was in accordance with the colocalization study.

In enterohepatic circulation, OST*α*/*β* is the main transporter that assists the exocytosis of bile acid in the basolateral side. The inhibitor of OST*α*/*β*, 17*α*‐ethinylestradiol, was used to study the connection between the transport of GCPN and OST*α*/*β*.^[^ ^43^ ^]^ It was found that 17*α*‐ethinylestradiol did not affect the transcytosis of CPN and GCPN (Figure 6f), suggesting they did not transport via OST*α*/*β*. This reveals the different transport mechanisms of GCPN and free GCA.

### 2.5 The Chylomicron Pathway in the Transport of CPN and GCPN in Caco‐2 Cells

The chylomicron pathway is a typical route for fat absorption. In the duodenum, the dietary lipids were emulsified by bile acids to increase the accessibility of lipases and co‐lipases, which hydrolyze the lipids into fatty acids and glycerol. The hydrolyzed products form micelles with bile acids, and digested fat molecules are actively or passively taken up by enterocytes in the duodenum.^[^ ^44^ ^]^ Then the hydrolyzed products are esterified into lipids like triglyceride (TG) in the endoplasmic reticulum, packed into chylomicrons and phospholipids and cholesterol esters.^[^ ^45^ ^]^ After exocytosis from the basolateral side of enterocytes, chylomicrons are transported to the lacteal and enter the systemic circulation via the intestinal lymphatic system.^[^ ^46^ ^]^

As mentioned in Introduction section, chylomicron pathway is an alternative route for the uptake of bile acid‐based nanoparticles. To clarify the relationship between the chylomicron pathway and GCPN in the Caco‐2 cell model, the inhibitor of chylomicron secretion, cycloheximide, was used. As shown in Figure S4a in the Supporting Information, cycloheximide reduced the transcytosis of GCPN but did not affect CPN transport across the Caco‐2 monolayers. Meanwhile, to further mimic the in vivo lipid transport, oleic acids and glycerol were added to the Caco‐2 cells as the hydrolyzed lipid. As shown in Figure S4b–e in the Supporting Information, with unknown mechanisms, the uptake of lipids and secretion of chylomicron were enhanced by GCPN as free bile acids did. It is also noted that ER‐Golgi route participates in the control of lipid droplets and the formation of chylomicron secretion,^[^ ^47^ ^]^ which was also utilized by GCPN transport. Thus, the transport of GCPN shared the chylomicron pathway. This discovery built up a connection between the ASBT‐mediated transport and the chylomicron pathway, which were considered separate before. On the one side, it has been validated that GCPN demonstrated ASBT‐mediated transport pathway, which is generally regarded in the ileum. On the other side, the chylomicron formation was mainly in the duodenum. The whole transcytosis process of GCPN was a new pathway that combined the transport characteristics of ASBT‐mediated pathway and chylomicron pathway.

### 2.6 Genomics Analysis of CPN and GCPN Transport

From the results above, the discrepancy between GCPN and CPN in transcytosis was smaller compared with the endocytosis study (approximately two‐ to three‐fold versus approximately five‐ to six‐fold). This indicates that GCPN had a higher intracellular retention ratio than CPN. The two NPs had different transcytosis routes, despite the similarities in the colocalization and transcytosis inhibition study. Various mechanisms might account for this phenomenon, such as the uptake of GCPN from the basolateral side, lower exocytosis of GCPN, or total alteration of the transport pathway. Therefore, we went further by investigating the genomic properties for deeper understanding.

**Figure 7:**
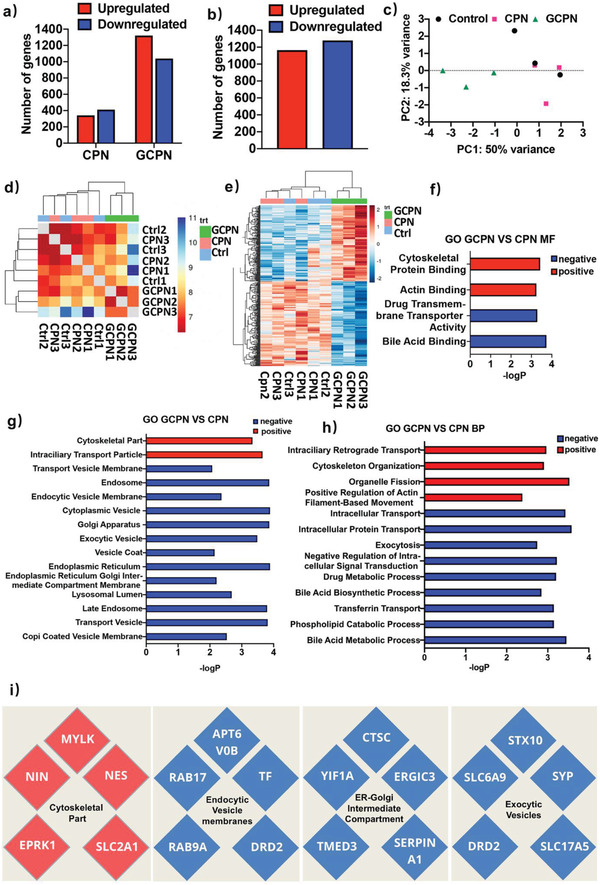
Genomics study of CPN and GCPN transport in Caco‐2 cells. a) Compared with control group, numbers of up‐ and downregulated genes after treatment with CPN and GCPN in 24 h. b) Compared with CPN group, number of up‐ and downregulated genes in GCPN group. c) PCA plot of the sample distribution. d) Sample Euclidean distance among the control, CPN, and GCPN. e) Heat map of the top 484 up‐ and downregulated genes in GCPN versus CPN. f–h) The statistical ORT based on the GO classification between GCPN versus CPN. The *p*‐values of the listed genes classes were all <0.05. i) Top upregulated (red) and downregulated (blue) genes in the corresponding pathways.

To better evaluate the interactions between the NPs and Caco‐2 cells at a molecular level, the gene expression after NPs treatment was studied by genomics method. More than 24 000 genes were traced. As shown in **Figure** **7**a,b, higher expression variety was observed after treatment with GCPN compared with CPN, with larger numbers of both upregulated and downregulated genes. In the principal components analysis (PCA) plot (Figure 7c), CPN showed very small variance with the control group, while GCPN plots were clustered away from the control and CPN. A similar result was observed in sample Euclidean distance (Figure 7d). It was clear that GCPN was further correlated with the other two groups, with the independent branches and the larger distance to the others. This illustrated that GCPN caused more specific gene expression regulation, revealing the GCA modification altered the Caco‐2 response to the NPs. The heat map of 484 selected significantly up‐ or downregulated genes, shown in Figure 7e, is consistent with the sample distance result. The top 10 upregulated genes in CPN versus Control, GCPN versus Control, and GCPN versus CPN are listed in Table S5 in the Supporting Information.

To explore the particular behaviors of CPN and GCPN, an overrepresentation test was performed based on the gene ontology (GO) strategy. This was classified into molecular function, cellular component, and biological process. The pathways significantly different between CPN and GCPN groups are summarized, and those relevant to the transcytosis are listed in Figure 7f–h. The lower *p*‐value (higher −log*P* value) represents greater difference between the GCPN and CPN groups, indicating higher expression in upregulated series and lower in downregulated ones.^[^ ^48^ ^]^ It was found that the bile acid binding (GO:0032052), bile acid biosynthetic process (GO:0006699), and bile acid metabolic process (GO:0008206) are all downregulated. It has been reported that bile acid synthesis is mainly controlled in transcriptional feedback regulation mode.^[^ ^49^ ^]^ In the chylomicron route study above, GCPN induced chylomicron production and secretion, which meant Caco‐2 cells responded to them similarly to GCA, leading to the downregulation of bile acid‐related routes. Analogously, lipid metabolism was also regulated in a feedback manner,^[^ ^50^ ^]^ and the phospholipid catabolic process (GO:0009395) was negatively regulated due to the higher uptake of digested lipids.

Compared with CPN, GCPN required enriched involvement of cytoskeleton for transport, with positively regulated pathways including cytoskeletal protein binding (GO:0008092), actin binding (GO:0003779), cytoskeletal part (GO:0044430), cytoskeleton organization (cytoskeleton organization), and positive regulation of actin filament‐based movement (GO:1903116). Intraciliary trafficking, a transport dependent on the microtube,^[^ ^51^ ^]^ was also activated with upregulated pathways: intraciliary retrograde transport (GO: 0035721) and intraciliary transport particle (GO:0030990). Although the uptake of both CPN and GCPN could be inhibited by CytD, their disparate interaction with Caco‐2 cells resulted in the different participation degree of actin, as ASBT was also internalized and recycled during the GCPN transport. The enhanced involvement of the cytoskeleton might be the response to the ASBT internalization requirement, as was the case with some other transporters.^[^ ^52^, ^53^ ^]^

Unlike the cytoskeleton‐dependent process, there were a number of transport pathways downregulated with the treatment of GCPN. Vesicle coat (GO:0030120) and transferrin transport (GO:0033572) were concerned with clathrin‐mediated endocytosis.^[^ ^54^ ^]^ Downregulation of the two pathways in GCPN group was attributed to the irrelevance of GCPN to clathrin‐mediated transport. The endosomal maturation process, including late endosome (GO:0005770) and lysosomal lumen (GO:0043202), were negatively regulated in GCPN group as well. Although it has been confirmed that the effect of the acidification process in CPN or GCPN transport was limited in colocalization and transcytosis study, CPN still exhibited higher relevance to the lysosomal degradation than GCPN (Figure 4). This was consistent with some previous research claiming that the ASBT‐targeting nano‐formulations were likely to escape from the lysosomes.^[^ ^20^, ^55^, ^56^ ^]^

The GCPN pathways relevant to the whole transport flow were largely and negatively regulated compared with those of CPN, such as endosome (GO:0005768), transport vesicle membrane (GO:0030658), intracellular transport (GO:0046907), intracellular protein transport (GO: 0006886), endoplasmic reticulum (GO:0005783), endoplasmic reticulum Golgi intermediate compartment membrane (GO:0033116), Golgi apparatus (GO:0005794), exocytosis (GO:0006887), and exocytic vesicles (GO:0070382). This meant that Caco‐2 cells responded to the GCPN in a negative feedback mode; in other words, Caco‐2 cells tended to inhibit their cargo intracellular transport ability with the treatment of GCPN. As shown in Figure 7i, the gene expression corresponding to the pathways was regulated differently with the treatment of CPN and GCPN, revealing their unique regulatory mechanisms for transcytosis. This accounted for the relatively lower transcytosis efficacy of GCPN, though GCPN showed higher uptake.

The in vivo genomics study was also performed in Balb/c mice. About 21 000 genes were traced and the result is shown in Figure S5 in the Supporting Information. GCPN group exhibited higher gene expression variety than CPN group to the control (Figure S5a,b, Supporting Information). The PCA plot, sample Euclidean distance, and heat map (of 500 genes) are shown in Figure S5c–e in the Supporting Information. The top 10 upregulated genes in CPN versus Control, GCPN versus Control, and GCPN versus CPN are listed in Table S6 in the Supporting Information. The GO analysis of the transport‐related pathways is shown in Figure S5f–h in the Supporting Information. Compared with the in vitro study, some difference was observed, like the upregulated organelle fission (GO:0048285) pathway and downregulated actin filament (GO:0005884) pathway by GCPN. Nevertheless, the major regulation mode was very similar: most of the transport pathways were downregulated, covering the whole transport flow: endocytic vesicle (GO:0030139), ER to Golgi transport vesicle membrane (GO:0012507), exocytic vesicle (GO:0070382), transporter complex (GO:1990351), etc. This is consistent with the in vitro study and further suggests the negative feedback to the GCPN transport. Figure S5i in the Supporting Information also shows the gene expression corresponding to the pathways was regulated differently with the treatment of CPN and GCPN, revealing their unique regulatory mechanisms for transcytosis.

Multiple studies were performed to validate this negative feedback of transport led by GCPN (Section S1, Supporting Information). The most straightforward method was the exocytosis study. Figure S6 in the Supporting Information displays the exocytosis of CPN and GCPN in Caco‐2 cells in 3 h, where CPN demonstrated higher excavating ratio (68.4% vs 55.4%). Besides, coumarin‐6 loaded micelles (C6‐M) were prepared and incubated with Caco‐2 cells which had been treated with CPN or GCPN for 24 h. The size of C6‐M was about 20 nm, and their uptake decreased once the Caco‐2 cells were treated with GCPN, while no difference was shown after CPN treatment (Figure S7, Supporting Information). These findings indicated that GCA modification triggered the negative feedback loop where the transport‐associated genes were downregulated and thus hampered the administration of the following nano‐formulations as well as slowed down the delivery of intracellular cargos. This means the higher oral bioavailability of GCPN was due to its ASBT‐mediated uptake, but the whole transcytosis efficacy was compensatorily weakened by negative regulation of transport pathways compared with endocytosis.

Meanwhile, the basolateral to apical (B to A) transport was also assayed to address the lower transcytosis efficiency of GCPN (Supporting Information S2). It was found that B to A transcytosis rate of GCPN was lower than that of CPN (Figure S8, Supporting Information). As ASBT was mainly expressed in the apical side of Caco‐2 monolayers (Video S4, Supporting Information), the B to A transcytosis of GCPN was nonspecific, and the difference of B to A transcytosis between CPN and GCPN mainly resulted from their surface properties. With the higher B to A uptake, CPNs were expected to demonstrate lower A to B transcytosis, which was contradictory to the previous results. Therefore, the lower A to B transcytosis ratio of GCPN was not owing to the B to A reabsorption but its negative regulation of the transport pathways. In addition, the basolateral NPs could be rapidly cleared by lymphatic vessels in vivo, which indicated that the B to A reabsorption had little influence on CPN and GCPN transcytosis in realistic conditions.

In a previous study, transferrin‐functionalized nanogranules were proven to stimulate the positive feedback loop in Caco‐2 monolayers, with no influence in Madin‐Darby Canine Kidney (MDCK).^[^ ^48^ ^]^ This was led by the different distribution of transferrin receptors in the two monolayers, with expression on both sides of Caco‐2 but only on the apical side of the MDCK. Besides, the bovine serum albumin (BSA)‐modified nanogranules did not affect transport regulation in the two monolayers. Similarly, our work revealed a point that is usually neglected in oral drug delivery study: transport pathways of the drugs is the outcome of bidirectional interaction of the drugs and the cells, which triggers systematic regulation in cells, rather than simply ligand–receptor interaction or one‐directional response of “smart” materials to the cellular microenvironment. To develop more effective oral nanomedicines, a macroscopic view of the whole transport regulation (such as feedback loop) is required, as well as the basic understanding of the specific interactions (like GCA‐ASBT).

## 3 Conclusion

**Figure 8:**
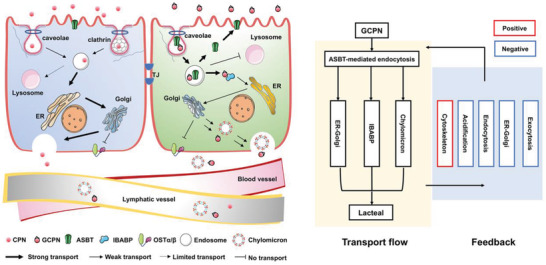
Schematic illustration of transport pathway regulation effect of GCA conjugation in Caco‐2 cells. Both NPs transported through the monolayer by transcytosis rather than paracellular pathway. Endocytosis of GCPN was mediated by ASBT and thus demonstrated stronger efficacy, which was assisted by caveolae. The uptake of CPN was nonspecific and mediated by clathrin and caveolae. After internalization, ASBT and GCPN were separated, with GCPN recycled to the membrane while GCPNs were bound to IBABP. Neither the two NPs was largely transported to lysosomes, and GCPN exhibited even less involvement in acidification. Both NPs transported through the ER‐Golgi route, while GCPN demonstrated lower efficacy in this pathway. After ER‐Golgi packaging, GCPN shared the chylomicron pathway and entered the lacteal after exocytosis. Neither of the NPs was transported via OST*α*/*β*. With GCA conjugation, the cells responded to the GCPN transport flow in a feedback mode, with cytoskeleton‐related pathway upregulated, while most transport progresses including the acidification, endocytosis, intracellular transport, and exocytosis were downregulated, which finally formed into a negative feedback loop.

This study sought to clarify the absorption mechanisms of bile acid transporter (ASBT)‐targeted nanomedicine using GCPN as a model nanoparticle and Caco‐2 cell line as the model enterocytes. The data tells a story of the complex connection between bile acid transporters, chylomicron transport pathway, and the underlying negative feedback transport regulation loop. As shown in **Figure** **8**, both CPNs and GCPNs were transported through transcytosis but not through paracellular pathways. Their endocytosis was in caveolae‐mediated pathway with the assistance of dynamin and actin filaments. With GCA modification, GCPN transported through ASBT‐mediated pathway, internalized together with ASBT, but not relied on clathrin which is involved in most receptor‐mediated endocytosis. After uptake, GCPNs were separated with ASBT and bound to IBABP. ASBT was gradually recycled back to the apical membrane. Both NPs were transported via the ER‐Golgi route and showed limited colocalization with lysosomes, and GCPN showed even less involvement in acidification. In ER‐Golgi, GCPN shared the chylomicron pathway and was transported into the lacteal after exocytosis.

Notably, compared with free bile acids, GCPN transport also showed relevance to ASBT and IBABP. They were also downregulating bile acid's synthetic and metabolic processes as free bile acids do. However, they were also delivered in the chylomicron pathway, which is solely for fat particles and mainly occurs in the duodenum. Besides, no involvement of OST*α*/*β* was observed. This was a clear indication that GCA modification maintained some properties of the bile acid ligand but also altered the remaining transport route from the free bile acid route into the chylomicron pathway. GCPN was transported through a new route to utilize the advantage of increased ASBT‐mediated cellular uptake and chylomicron transport.

It should be mentioned, however, that the transport flow of GCPN could also trigger the response of Caco‐2 cells, which enhanced their actin activity and subsequently the intraciliary transport, ultimately downregulating almost all the transport stages including critical intracellular transport, exocytosis, and acidification process. This may account for the relatively lower GCPN transcytosis efficacy than their uptake, and the higher transmembrane amount of GCPN was due to the larger base of the endocytosis. Meanwhile, limitations in the chosen cell models, such as using the colon cancer cell line Caco‐2, could also qualify these conclusions. These conundrums serve to remind researchers that the design of bile acid‐based nanomedicine requires a comprehensive understanding of the whole transport process. In future studies, great care must be taken to choose appropriate technologies to regulate each distinct transport mechanism. Besides, it is noted that the prerequisite of this mechanism study is that the solid GCPN is stable in the gastrointestinal (GI) tract. For the application of bile acid‐modified nanotechnology in oral delivery of BCS III/IV drugs, the integrity of the carrier is essential to keep the active pharmaceutical ingredients from the harsh GI environment. Therefore, some stabilizing technologies, like polymer coating or bilosomes,^[^ ^13^ ^]^ are required for the ASBT‐mediated chylomicron pathway of bile acid‐modified nanomedicines. Overall, this study provides a foundation and insight into oral bile acid‐based nanodrug design, hopefully accelerating their development to clinical application and overcoming obstacles of low oral bioavailability drugs.

## 4 Experimental Section

### Materials

GCA, cycloheximide, and CFL488 labeled secondary antibody were purchased from Santa Cruz (Dallas, TX, USA). CPN (100 nm, carboxylate, fluorescence *E*_x_ = 552 nm, *E*_m_ = 580 nm) was ordered from Micromod Partikeltechnologie GmbH (Rostock, Germany). Dynasore and ZCL278 were provided by Abmole Bioscience (Houston, TX, USA). Nystatin, genistein, EIPA, CPZ, oleic acid, Sudan Red 7B, glycerol, 17*α*‐ethinylestradiol, and siRNAs of RhoA, Arf6, and Flot‐1 were purchased from Sigma Aldrich (St Louis, Mo, USA). Brefeldin and triglyceride colorimetric assay kit were obtained from Cayman Chemical (Ann Arbor, MI, USA). Bafilomycin was ordered from StressMarq Bioscience (Victoria, British Columbia, Canada). The primers for qPCR analysis were synthesized by Eurofins Genomics (Louisville, KY, USA). The antibodies of ASBT and Rab7 were provided by Abcam (Cambridge, MA, USA) and Bioss Antibodies (Woburn, MA, USA) respectively. Endoplasmic reticulum and Golgi apparatus staining kits were obtained from Abcam (Cambridge, MA, USA). Lysotracker green DND‐26 and Trizol were ordered from Invitrogen (Carlsbad, CA, USA). Human ApoB100 ELISA kit was purchased from Thermofisher Scientific (Carlsbad, CA, USA). JetPRIME was ordered from Polyplus Transfection (New York, NY, USA). Direct‐zol RNA MiniPrep was purchased from Zymo Research (Irvine, CA, USA). Transwells (12 wells, 3 µm pore size, polycarbonate membranes) were provided by Corning Inc. Costar (Cambridge, MA, USA). The Caco‐2 cell line was obtained from American Type Culture Collection (ATCC, Manassas, VA, USA). Balb/c mice were purchased from the Jackson Laboratory (Bar Harbor, ME, USA).

### Preparation and Characterization of GCPN

GCPN was synthesized according to previous work.^[^ ^21^ ^]^ Briefly, 300 mg GCA was dissolved in 3 mL dimethylformamide. *N*,*N*′‐dicyclohexylcarbodiimide (DCC) (173 mg), *N*‐hydroxysuccinimide (NHS) (96 mg), and ethylenediamine (EDA) (2.15 mL) were added to the solution, and stirred overnight at room temperature. After the reaction, EDA was removed by evaporation at 80 °C and the product was washed with ethyl acetate. The precipitation pellet (GCA‐EDA) was dissolved in water, filtered, and lyophilized. To synthesize GCPN, CPN (1 mg) was dispersed in 0.1 m 2‐(*N*‐morpholino)ethanesulfonic acid buffer (pH = 6.0, 2 mL) and activated with 1‐ethyl‐3‐(3‐(dimethylamino) propyl) carbodiimide hydrochloride (2.5 mg) and NHS (2.5 mg). Then, GCA‐EDA (5 mg) was added and stirred overnight at room temperature. The product was purified by ultracentrifugation at 3000 rpm for 10 min. The synthesis was confirmed by NMR (Mercury 400). The size and Zeta potential of CPN and GCPN were determined by dynamic light scattering using Malvern Zetasizer Nano ZS (Malvern, U.K.). The morphology of the NPs was observed by TEM (JEOL JEM‐1400).

### Endocytosis Study of CPN and GCPN in Caco‐2 Cells

Caco‐2 cells were seeded in 12‐well plates at 1 × 10^5^ per well. After the cells met 80% confluency, 100 µg mL^−1^ of CPN and GCPN were added incubated at 37 °C for 30 min. Then the cells were washed with cold phosphate‐buffered saline (PBS) three times and collected by trypsin. The cells were further rinsed and the intracellular fluorescence intensity was measured by flow cytometry (FACS Canto II, BD Biosciences, San Jose, CA, USA). Meanwhile, the cells after treatment were observed by CLSM (TCS SP8, Leica, Wetzlar, Germany).

### Endocytosis Pathway of CPN and GCPN in Caco‐2 Cells

For the study of the effect of GCA competition on CPN and GCPN endocytosis, Caco‐2 cells were seeded as described above. Then the cells were treated with 1 mg mL^−1^ free GCA for 1 h followed by 100 µg mL^−1^ of CPN and GCPN for another 0.5 h. The following procedure for flow cytometry analysis was the same as described above. For temperature effect, Caco‐2 cells were incubated with blank media at designated temperature (37, 20, or 4 °C) for 1 h. Then the cells were treated with precooled or prewarmed CPN and GCPN for another 0.5 h. The intracellular fluorescence was measured by flow cytometry.

The cytotoxicity of various pharmacological inhibitors (Table S1, Supporting Information) was explored by MTT assay. Briefly, 5 × 10^3^ per well of Caco‐2 cells were seeded in a 96‐well plate and treated with the inhibitors for 1 h at 37 °C. Then the cells were washed and treated with 100 µL of 0.5 mg mL^−1^ MTT at 37 °C for 4 h. The media was carefully removed and 100 µL dimethyl sulfoxide was added. The absorption was measured at UV 570 nm after 30 min incubation at 37 °C. To study the effect of the pharmacological inhibitors, 1 × 10^5^ per well cells were seeded in 12‐well plates and incubated with the inhibitors at 37 °C for 0.5 h. Then the cells were treated with GCPN or CPN together with the inhibitors for 0.5 h. The intracellular fluorescence intensity was measured with flow cytometry.

### Clathrin/Caveolae‐Independent Endocytosis Pathway Study

To study the expression of RhoA, Arf6, and Flot‐1 after knocking down, Caco‐2 cells were seeded in 6‐well plate at a density of 1.5 × 10^5^ per well. After the cells reached 50% confluency, siRNAs of RhoA, Arf6, and Flot‐1 were transfected to the cells with jetPRIME according to the manufacturer's instruction. Negative siRNA with no specific target was used as the control. After 48 h, the cells were washed with PBS and the RNA was extracted by Trizol kit according to the manufacturer's instruction. The cDNA was then synthesized using RNA (1 µg), dNTP, oligo (dT) 20‐mer, and reverse transcriptase. Then real‐time PCR analysis was performed by StepOnePlus real‐time PCR system (Applied Biosystem) with human *β*‐actin for gene expression normalization. The oligonucleotide primer sequences are shown in Table S3 in the Supporting Information.

To investigate the effect of RhoA, Arf6, Flot‐1, and Cdc42 on CPN and GCPN endocytosis, Caco‐2 cells were seeded in a 12‐well plate at 1 × 10^5^ per well. After the cell reached 50% confluency, siRNA of RhoA, Arf6, and Flot‐1 was transfected to the cells with jetPRIME according to the manufacturer's instruction. After 48 h, the cells were washed with PBS and treated with 100 µg mL^−1^ of CPN and GCPN and incubated for 0.5 h. For cdc42 effect exploration, the cells were treated with 50 × 10^−6^ m ZCL278 followed by ZCL278 with the NPs. The intracellular fluorescence intensity was measured with flow cytometry.

### ASBT Distribution after CPN and GCPN Treatment

Caco‐2 cells were seeded on glass slides in a 24‐well plate and treated with 200 µg mL^−1^ of CPN and GCPN for 2, 8, and 24 h, respectively. After treatment, the cells were washed three times with PBS and fixed with 4% paraformaldehyde for 15 min. The unspecific binding was blocked by incubation with 1% BSA at 37 °C for 30 min and the cell nuclei were stained with Hoechst 33258 for 30 min. ASBT was stained with primary antibody at 4 °C overnight and CFL488 labeled secondary antibody at 37 °C for 2 h. The samples were observed by CLSM.

### Colocalization of Intracellular Organelles and CPN/GCPN

Caco‐2 cells were seeded on glass slides in a 24‐well plate and treated with 200 µg mL^−1^ of CPN and GCPN for 1 h. Then ER, Golgi apparatus, and lysosomes were stained with ER staining kit, Golgi apparatus staining kit, and lysotracker green DND‐26 according to the manufacturer's instruction, respectively. Late endosomes were stained with primary antibody at 4 °C overnight and CFL488 labeled secondary antibody at 37 °C for 2 h after the cells were fixed with 4% paraformaldehyde, permeabilized with 0.1% TritonX‐100, and blocked with 1% BSA. The samples were observed by CLSM. 15 areas were selected randomly to calculate the average Pearson's coefficient. Cells with no NPs added were used as negative control.

### IBABP Expression and Colocalization Analysis after CPN and GCPN Treatment

Caco‐2 cells were seeded in a 6‐well plate and treated with 200 µg mL^−1^ of CPN and GCPN for 24 h. After treatment, the cells were washed with PBS. The RNA extraction, cDNA synthesis, and RT‐PCR analysis steps were the same as described above. The oligonucleotide primer sequence is in Table S3 in the Supporting Information. For colocalization analysis, Caco‐2 cells were treated with 200 µg mL^−1^ CPN and GCPN for 3 h. Then the cells were fixed with 4% paraformaldehyde, permeabilized with 0.1% TritonX‐100, and blocked with 1% BSA. The cells were treated with primary antibody of IBABP at 4 °C overnight and CFL488 labeled secondary antibody at 37 °C for 2 h. The samples were observed by CLSM.

### Caco‐2 Monolayer Culture

Caco‐2 cells were seeded in the transwell filters at a 2 × 10^5^ per well density and allowed to grow and differentiate. The media was refreshed every 2 days in the first week and every day since the second week. The cell monolayers were used when the TEER exceeded 400 Ω cm^2^.

### Paracellular Transport Pathway of CPN/GCPN across the Monolayer

Paracellular pathway was tested by evaluating the integrity of tight junctions in the Caco‐2 monolayers. In brief, the media containing 10 µg mL^−1^ fluorescein sodium (Na‐Flu) and 500 µg mL^−1^ CPN or GCPN were added to the apical chamber of the monolayer and incubated at 37 °C. At predetermined time points (0, 1, 2, 4, 8, and 12 h), the TEER was measured, and 100 µL media were collected from basolateral chambers where another 100 µL of fresh media were added. The fluorescence intensity was measured by the microplate reader at *E*_x_ = 493 nm, *E*_m_ = 520 nm (CYTATION3, BioTek, USA). The accumulative amount of fluorescein sodium was calculated using a standard curve. The cells treated with 0.5% chitosan (w/v) with Na‐Flu was used as the positive control and those with only Na‐Flu treatment as the negative one.

### Transcytosis Analysis of CPN and GCPN across the Caco‐2 Monolayer

After the cell monolayers were ready to use, 500 µg mL^−1^ CPN and GCPN were added to the apical chamber of the monolayer and incubated at 37 °C. At predetermined time points (0, 6, and 12 h), the TEER was measured, and 100 µL media were collected from basolateral chambers where another 100 µL of fresh media were added. The fluorescence intensity was measured by a microplate reader (BioTek, USA). To observe the distribution of CPN and GCPN, the cells were incubated with CPN or GCPN for 12 h, washed with PBS, and fixed by 4% paraformaldehyde for 15 min. The nuclei were stained with Hoechst 33258 for 30 min. Then the membrane was carefully cut down, placed upside down on a glass‐bottom culture dish (35 mm dish with 20 mm glass bottom well) with 50 µL PBS between the cell monolayer and the glass bottom. The monolayer was scanned by CLSM and the 3D model was recorded.

### Transcytosis Pathway of CPN and GCPN across the Caco‐2 Monolayer

After the cell monolayers were ready to use, the monolayers were treated with various transcytosis inhibitors (Table S4, Supporting Information) for 1 h. Then the monolayers were incubated with 500 µg mL^−1^ CPN and GCPN together with the inhibitors at 37 °C for another 12 h. The TEER was measured at predetermined time points (0, 3, 6, and 12 h). After treatment, 100 µL media were collected from basolateral chambers and the fluorescence intensity was measured by the microplate reader.

### Effect of Chylomicron Secretion Inhibition on the Transcytosis of CPN and GCPN across the Caco‐2 Monolayer

After the cell monolayers were ready to use, the monolayers were treated with 50 µg mL^−1^ cycloheximide for 1 h. Then the monolayers were incubated with 500 µg mL^−1^ CPN and GCPN together with the 50 µg mL^−1^ cycloheximide at 37 °C for another 12 h. After treatment, 100 µL media were collected from basolateral chambers and the fluorescence intensity was measured by the microplate reader.

### Interaction between CPN/GCPN and Chylomicron

After the cell monolayers were ready to use, the monolayers were treated with i) 2 × 10^−3^ m oleic acid + 2 × 10^−3^ m glycerol + 200 µg mL^−1^ CPN; ii) 2 × 10^−3^ m oleic acid + 2 × 10^−3^ m glycerol + 200 µg mL^−1^ GCPN in phenol red‐free media, and then incubated at 37 °C for 24 h. Then the basolateral media were collected and mixed with 6 mL NaCl (1.25 g mL^−1^) in centrifuge tubes. The mixture was overlaid with 0.5 mL water and centrifuged at the speed of 10 000 rpm at 4 °C for 30 min. The 100 µL of the upper layer was collected and the fluorescence intensity was measured by the microplate reader.

### Triglyceride (TG) and Apolipoprotein B (ApoB) Secretion with CPN and GCPN Treatment in Caco‐2 Cells

Caco‐2 cells were seeded in 100 mm culture dish and allowed to grow for 7 days. Then the cells were treated as follows and incubated at 37 °C for 4 h separately: i) 2 × 10^−3^ m oleic acid + 2 × 10^−3^ m glycerol; ii) 2 × 10^−3^ m oleic acid + 2 × 10^−3^ m glycerol + 1 × 10^−3^ m GCA; iii) 2 × 10^−3^ m oleic acid + 2 × 10^−3^ m glycerol + 200 µg mL^−1^ CPN; iv) 2 × 10^−3^ m oleic acid + 2 × 10^−3^ m glycerol + 200 µg mL^−1^ GCPN. After treatment, the media were removed, followed by 4 mL phenol red‐free media added and incubation at 37 °C for another 2 h. Then the media was mixed with 16 mL NaCl (1.25 g mL^−1^) and 0.1 mL Fat Red 7B (in 0.1 m NaOH solution with 5 µL TritonX‐100) in centrifuge tubes. The mixture was overlaid by 2 mL water and centrifuged at the speed of 10 000 rpm at 4 °C for 30 min. The 500 µL of the upper layer with Fat Red 7B staining was collected. The TG and ApoB concentrations were determined by triglyceride colorimetric assay kit and human ApoB100 ELISA kit according to the manufacturer's instruction. Meanwhile, the cells were lysed by 600 µL radioimmunoprecipitation assay buffer and the protein concentration was measured by bicinchoninic acid test. The TG and ApoB secretion efficacies were normalized by the ratio of TG and ApoB concentration to the corresponding protein concentration.

### Intracellular Chylomicron Staining

Caco‐2 cells were seeded in 6‐well plates and allowed to grow for 7 days. Then the cells were treated as described above. After treatment, the cells were washed and harvested by trypsin. Then the cells were stained with 0.25 µg mL^−1^ Nile Red for 30 min. The samples were observed by CLSM at the dual excitation wavelength 450 and 515 nm, and the wavelength reception range was 530–550 nm.

### Genomics Study of CPN and GCPN Transport in Caco‐2 Cells

Caco‐2 cells were seeded in 6‐well plates. At 80% confluency, the cells were treated with 200 µg mL^−1^ CPN and GCPN for 24 h. Then the cells were washed with PBS and the RNA was extracted and purified by Trizol and Direct‐zol RNA MiniPrep according to the manufacturer's instruction. The RNA library was prepared and the sequencing was performed by Illumina Stranded Total RNA Library Prep w Ribo‐Zero Plus** to a depth of ≈20–40 million mapped reads per sample. For analysis of expression variation, reads were aligned to GRCh38/hg38 using Novoalign (Novocraft). Reads were normalized using DESeq2 analysis package. Heatmaps, clustering and principal component analysis were generated in R. Normalized spot intensities were transformed to gene expression log2 ratios between the different groups. Gene ontology enrichment was analyzed by DAVID.

### Genomics Study of CPN and GCPN Transport in Balb/c Mice

The animal experiment was approved by University of Utah's Animal Care and Use Committee (#protocol: 17–06003). Balb/c mice (8 weeks) were divided into three groups (*n* = 4 for each group). The mice were orally administrated with 10 mg kg^−1^ CPN or GCPN every day in 2 weeks. The control group mice were administrated with the same volume of PBS. Then the mice were sacrificed under anesthesia. The ileum was harvested and stored at −80 °C. Then the mRNA was extracted and the genomics analysis was performed with the same method in the genomics study of Caco‐2 cells.

### Statistical Analysis

The results are expressed as mean ± SD unless otherwise stated. All results were analyzed by analysis of variance (ANOVA) or *t*‐test unless specifically clarified. A *p*‐value less than 0.05 was considered statistically significant, while a *p*‐value less than 0.01 was considered to be highly significant.

## Conflict of Interest

The authors declare no conflict of interest.

## Supporting information

Supporting Information

Supplemental Video 1

Supplemental Video 2

Supplemental Video 3

Supplemental Video 4

## Data Availability

Research data are not shared.

## References

[1] S. Hua , Front. Pharmacol. 2020, 11, 524. 32425781 10.3389/fphar.2020.00524 PMC7212533

[2] B. Homayun , X. Lin , H. J. Choi , Pharmaceutics 2019, 11, 129. 10.3390/pharmaceutics11030129 PMC6471246 30893852

[3] S. J. Cao , S. Xu , H. M. Wang , Y. Ling , J. Dong , R. D. Xia , X. H. Sun , AAPS PharmSciTech 2019, 20, 190. 31111296 10.1208/s12249-019-1325-z PMC6527526

[4] H. Lennernas , B. Abrahamsson , E. M. Persson , L. Knutson , J. Drug Delivery Sci. Technol. 2007, 17, 237.

[5] S. Ganta , D. Deshpande , A. Korde , M. Amiji , Mol. Membr. Biol. 2010, 27, 260. 20929336 10.3109/09687688.2010.497971

[6] L. Gao , G. Y. Liu , J. L. Ma , X. Q. Wang , L. Zhou , X. Li , F. Wang , Pharm. Res. 2013, 30, 307. 23073665 10.1007/s11095-012-0889-z

[7] R. Kozyraki , J. Fyfe , M. Kristiansen , C. Gerdes , C. Jacobsen , S. Cui , E. I. Christensen , M. Aminoff , A. de la Chapelle , R. Krahe , P. J. Verroust , S. K. Moestrup , Nat. Med. 1999, 5, 656. 10371504 10.1038/9504

[8] J. Wang , J. Tan , J. Luo , P. Huang , W. Zhou , L. Chen , L. Long , L. M. Zhang , B. Zhu , L. Yang , D. Y. Deng , J. Nanobiotechnol. 2017, 15, 18. 10.1186/s12951-017-0251-z PMC5333415 28249594

[9] A. K. Petrus , T. J. Fairchild , R. P. Doyle , Angew. Chem., Int. Ed. Engl. 2009, 48, 1022. 19072807 10.1002/anie.200800865

[10] A. F. Hofmann , Arch. Intern. Med. 1999, 159, 2647. 10597755 10.1001/archinte.159.22.2647

[11] J. Y. Chiang , Compr. Physiol. 2013, 3, 1191. 23897684 10.1002/cphy.c120023 PMC4422175

[12] M. S. Roberts , B. M. Magnusson , F. J. Burczynski , M. Weiss , Clin. Pharmacokinet. 2002, 41, 751. 12162761 10.2165/00003088-200241100-00005

[13] F. Deng , Y. H. Bae , J. Controlled Release 2020, 327, 100. 10.1016/j.jconrel.2020.07.034 PMC7606772 32711025

[14] Z. Khatun , M. Nurunnabi , G. R. Reeck , K. J. Cho , Y. K. Lee , J. Controlled Release 2013, 170, 74. 10.1016/j.jconrel.2013.04.024 23665255

[15] K. Suzuki , K. S. Kim , Y. H. Bae , J. Controlled Release 2019, 294, 259. 10.1016/j.jconrel.2018.12.028 PMC6369909 30572033

[16] M. Nakahara , N. Furuya , K. Takagaki , T. Sugaya , K. Hirota , A. Fukamizu , T. Kanda , H. Fujii , R. Sato , J. Biol. Chem. 2005, 280, 42283. 16230354 10.1074/jbc.M507454200

[17] A. Rao , J. Haywood , A. L. Craddock , M. G. Belinsky , G. D. Kruh , P. A. Dawson , Proc. Natl. Acad. Sci. U. S. A. 2008, 105, 3891. 18292224 10.1073/pnas.0712328105 PMC2268840

[18] N. J. Hu , S. Iwata , A. D. Cameron , D. Drew , Nature 2011, 478, 408. 21976025 10.1038/nature10450 PMC3198845

[19] H. Westergaard , J. M. Dietschy , J. Clin. Invest. 1976, 58, 97. 932213 10.1172/JCI108465 PMC333160

[20] T. A. Al‐Hilal , S. W. Chung , F. Alam , J. Park , K. E. Lee , H. Jeon , K. Kim , I. C. Kwon , I. S. Kim , S. Y. Kim , Y. Byun , Sci. Rep. 2014, 4, 4163. 24566561 10.1038/srep04163 PMC3933907

[21] K. S. Kim , K. Suzuki , H. Cho , Y. S. Youn , Y. H. Bae , ACS Nano 2018, 12, 8893. 30088412 10.1021/acsnano.8b04315 PMC6377080

[22] H. A. Cognart , J. L. Viovy , C. Villard , Sci. Rep. 2020, 10, 6386. 32286431 10.1038/s41598-020-63316-w PMC7156718

[23] Y. Sambuy , I. De Angelis , G. Ranaldi , M. L. Scarino , A. Stammati , F. Zucco , Cell Biol. Toxicol. 2005, 21, 1. 15868485 10.1007/s10565-005-0085-6

[24] K. S. Kim , D. S. Kwag , H. S. Hwang , E. S. Lee , Y. H. Bae , Mol. Pharmaceutics 2018, 15, 4756. 10.1021/acs.molpharmaceut.8b00708 PMC6588159 30125508

[25] F. Alam , T. A. Al‐Hilal , S. W. Chung , D. Seo , F. Mahmud , H. S. Kim , S. Y. Kim , Y. Byun , Biomaterials 2014, 35, 6543. 24816287 10.1016/j.biomaterials.2014.04.050

[26] Y. Yu , M. Huo , Y. Fu , W. Xu , H. Cai , L. Yao , Q. Chen , Y. Mu , J. Zhou , T. Yin , Mol. Pharmaceutics 2017, 14, 4539. 10.1021/acs.molpharmaceut.7b00662 29058910

[27] L. M. Bareford , P. W. Swaan , Adv. Drug Delivery Rev. 2007, 59, 748. 10.1016/j.addr.2007.06.008 PMC2000329 17659804

[28] T. Kosaka , K. Ikeda , J. Cell Biol. 1983, 97, 499. 6411734 10.1083/jcb.97.2.499 PMC2112522

[29] N. L. Chanaday , E. T. Kavalali , FEBS Lett. 2018, 592, 3606. 30311950 10.1002/1873-3468.13268

[30] M. Kaksonen , A. Roux , Nat. Rev. Mol. Cell Biol. 2018, 19, 313. 29410531 10.1038/nrm.2017.132

[31] G. J. Doherty , H. T. McMahon , Annu. Rev. Biochem. 2009, 78, 857. 19317650 10.1146/annurev.biochem.78.081307.110540

[32] S. Y. Song , W. S. Cong , S. R. Zhou , Y. J. Shi , W. B. Dai , H. Zhang , X. Q. Wang , B. He , Q. Zhang , Asian J. Pharm. Sci. 2019, 14, 30. 32104436 10.1016/j.ajps.2018.06.004 PMC7032109

[33] A. Friesland , Y. Zhao , Y. H. Chen , L. Wang , H. Zhou , Q. Lu , Proc. Natl. Acad. Sci. U. S. A. 2013, 110, 1261. 23284167 10.1073/pnas.1116051110 PMC3557054

[34] Y. Tu , L. Zhao , D. D. Billadeau , D. Jia , Front. Cell Dev. Biol. 2020, 8, 163. 32258039 10.3389/fcell.2020.00163 PMC7093645

[35] N. Gomez‐Navarro , E. Miller , J. Cell Biol. 2016, 215, 769. 27903609 10.1083/jcb.201610031 PMC5166505

[36] Y. B. Hu , E. B. Dammer , R. J. Ren , G. Wang , Transl. Neurodegener. 2015, 4, 18. 26448863 10.1186/s40035-015-0041-1 PMC4596472

[37] S. T. Hwang , N. L. Urizar , D. D. Moore , S. J. Henning , Gastroenterology 2002, 122, 1483. 11984532 10.1053/gast.2002.32982

[38] T. Claudel , B. Staels , F. Kuipers , Arterioscler., Thromb., Vasc. Biol. 2005, 25, 2020. 16037564 10.1161/01.ATV.0000178994.21828.a7

[39] G. D. Amoutzias , E. E. Pichler , N. Mian , D. De Graaf , A. Imsiridou , M. Robinson‐Rechavi , E. Bornberg‐Bauer , D. L. Robertson , S. G. Oliver , BMC Syst. Biol. 2007, 1, 34. 17672894 10.1186/1752-0509-1-34 PMC1971058

[40] J. Smith , E. Wood , M. Dornish , Pharm. Res. 2004, 21, 43. 14984256 10.1023/b:pham.0000012150.60180.e3

[41] T. Fujiwara , K. Oda , S. Yokota , A. Takatsuki , Y. Ikehara , J. Biol. Chem. 1988, 263, 18545. 3192548

[42] Y. M. Yan , K. Jiang , P. Liu , X. B. Zhang , X. Dong , J. C. Gao , Q. T. Liu , M. P. Barr , Q. Zhang , X. K. Hou , S. S. Meng , P. Gong , Sci. Rep. 2016, 6, 37052. 27845389 10.1038/srep37052 PMC5109251

[43] M. M. Malinen , A. Kauttonen , J. J. Beaudoin , N. Sjostedt , P. Honkakoski , K. L. R. Brouwer , Mol. Pharmaceutics 2019, 16, 238. 10.1021/acs.molpharmaceut.8b00966 PMC6465078 30481467

[44] J. Iqbal , M. M. Hussain , Am. J. Physiol. Endocrinol. Metab. 2009, 296, E1183. 19158321 10.1152/ajpendo.90899.2008 PMC2692399

[45] C. W. Ko , J. Qu , D. D. Black , P. Tso , Nat. Rev. Gastroenterol. Hepatol. 2020, 17, 169. 32015520 10.1038/s41575-019-0250-7

[46] J. B. Dixon , Ann. N. Y. Acad. Sci. 2010, 1207, E52. 20961306 10.1111/j.1749-6632.2010.05716.x PMC3132563

[47] D. Hesse , A. Jaschke , B. Chung , A. Schurmann , Biosci. Rep. 2013, 33, e00001. 23033902 10.1042/BSR20120082 PMC3522472

[48] D. Yang , D. Liu , H. Deng , J. Zhang , M. Qin , L. Yuan , X. Zou , B. Shao , H. Li , W. Dai , H. Zhang , X. Wang , B. He , X. Tang , Q. Zhang , ACS Nano 2019, 13, 5058. 31034211 10.1021/acsnano.8b07231

[49] T. Li , J. Y. Chiang , J. Lipids 2012, 2012, 754067. 21991404 10.1155/2012/754067 PMC3185234

[50] C. J. Loewen , M. L. Gaspar , S. A. Jesch , C. Delon , N. T. Ktistakis , S. A. Henry , T. P. Levine , Science 2004, 304, 1644. 15192221 10.1126/science.1096083

[51] Q. Wei , K. Ling , J. Hu , Curr. Opin. Cell Biol. 2015, 35, 98. 25988548 10.1016/j.ceb.2015.04.015 PMC4529799

[52] L. R. Gabriel , S. Wu , P. Kearney , K. D. Bellve , C. Standley , K. E. Fogarty , H. E. Melikian , J. Neurosci. 2013, 33, 17836. 24198373 10.1523/JNEUROSCI.3284-13.2013 PMC3818556

[53] E. Penalver , L. Ojeda , E. Moreno , R. Lagunas , Yeast 1997, 13, 541. 9178505 10.1002/(SICI)1097-0061(199705)13:6<541::AID-YEA112>3.0.CO;2-4

[54] K. M. Mayle , A. M. Le , D. T. Kamei , Biochim. Biophys. Acta 2012, 1820, 264. 21968002 10.1016/j.bbagen.2011.09.009 PMC3288267

[55] S. W. Wu , W. Bin , B. Y. Tu , X. F. Li , W. Wang , S. L. Liao , C. S. Sun , J. Pharm. Sci. 2019, 108, 2143. 30721709 10.1016/j.xphs.2019.01.027

[56] W. Fan , D. Xia , Q. Zhu , X. Li , S. He , C. Zhu , S. Guo , L. Hovgaard , M. Yang , Y. Gan , Biomaterials 2018, 151, 13. 29055774 10.1016/j.biomaterials.2017.10.022

