# Supplementary material for: Bile Acid Conjugation on Solid Nanoparticles Enhances ASBT‐Mediated Endocytosis and Chylomicron Pathway but Weakens the Transcytosis by Inducing Transport Flow in a Cellular Negative Feedback Loop
Source: Adv Sci (Weinh). 2022 Jun 2;9(21):2201414. doi: 10.1002/advs.202201414 (PMC9313510; doi:10.1002/advs.202201414)
Supplement: Supplementary file 1 — Supporting Information [file ADVS-9-2201414-s002.pdf]

Supporting Information

Bile acid conjugation on solid nanoparticles enhances ASBT-mediated endocytosis and chylomicron pathway but weakens the transcytosis by inducing transport flow in a cellular negative feedback loop

*Feiyang Deng, Kyoung Sub Kim, Jiyoung Moon and You Han Bae**

Department of Pharmaceutics and Pharmaceutical Chemistry
College of Pharmacy, University of Utah
30 S 2000 E, Salt Lake City, Utah, 84112, USA

E-mail: you.bae@utah.edu

S1. Verification of the transport flow feedback by micelles

S1.1. Exocytosis of CPN and GCPN in Caco-2 cells

Caco-2 cells were seeded in a 12-well plate at 1×10^5/well and treated with 100 μg/mL of CPN and GCPN for 0.5 h. Then the cells were washed thoroughly with PBS for 3 times, and incubated with blank media for exocytosis for another 3 h. The cells were washed and the intracellular fluorescence was measured by flow cytometry as described in Section S2.1.1. The cells that did not go through the exocytosis step were used as the control.

S1.2 Preparation and characterization of Coumarin-6 loaded micelles

Coumarin-6 loaded micelles were prepared in the following procedures: DSPE-PEG and coumarin-6 were dissolved in acetonitrile in a round-bottom flask at the ratio of 1000:1 (w/w). The solution was evaporated into a film at 60 °C for 30 min. Then the film was

hydrated with 2 mL PBS at 60 °C for 5 min, sonicated in water-bath for 5 min and probe-sonicated for 5 min. The size of the micelles was measured by DLS.

S1.3 Endocytosis of Coumarin-6 loaded micelles

Caco-2 cells were seeded in a 12-well plate at 1×10^5/well and treated with 100 μg/mL of CPN and GCPN for 24 h. Then the cells were washed with PBS and treated with 500 μg/mL coumarin-6-loaded micelles for 0.5 h. The cells were then harvested and the intracellular fluorescence was measured by flow cytometry in the FITC channel. The images of the samples were also taken by CLSM. The cells treated with CPN and GCPN only were used to eliminate the background.

S2. Transcytosis of CPN and GCPN from basolateral to apical side (B to A) across Caco-2 monolayers

After the cell monolayers were ready to use, ASBT distribution was observed by CLSM with immunofluorescence labeling. To test the B to A transport of the NPs, 500 μg/mL CPN and GCPN were added to the basolateral chamber of the monolayer and incubated at 37 °C. After 24 h, 100 μL media were collected from basolateral chambers. The fluorescence intensity was measured by a microplate reader.

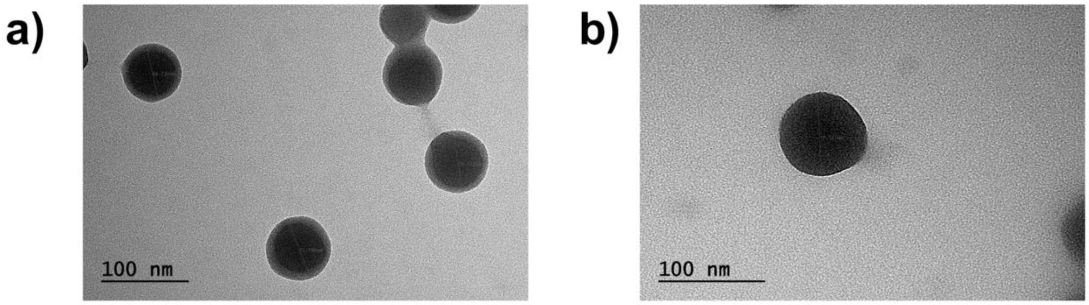

Figure S1. TEM images of a) CPN and b) GCPN.

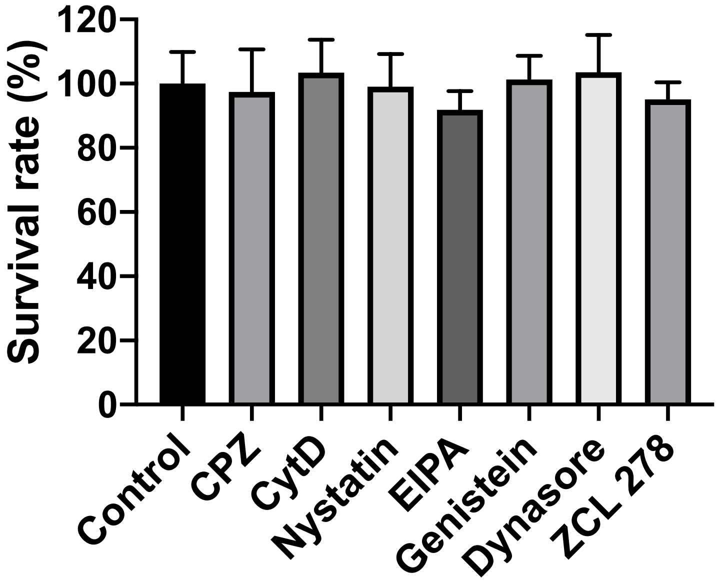

Figure S2. Cytotoxicity of various pharmacological inhibitors in Caco-2 cell line.

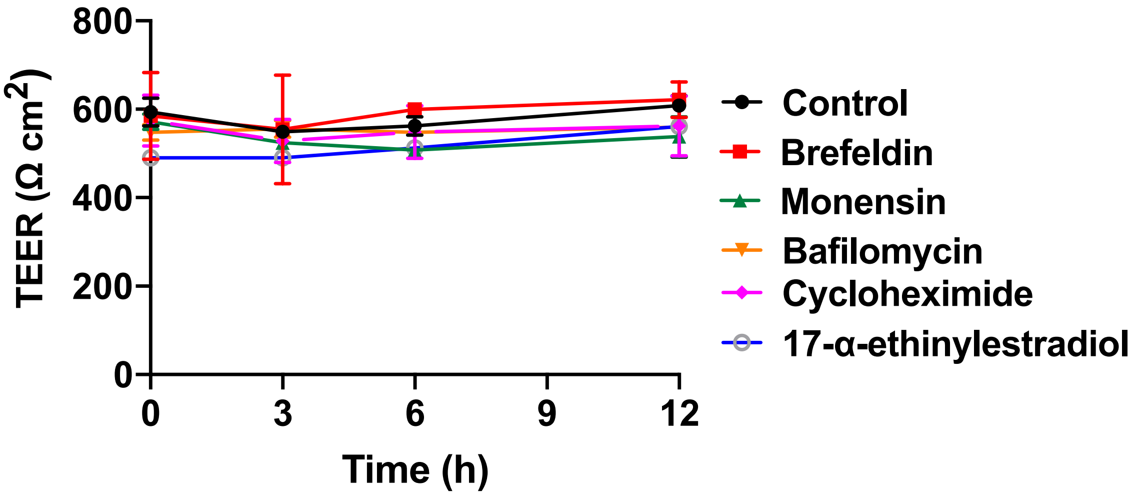

Figure S3. TEER values of Caco-2 monolayers incubated with various inhibitors in 12 h.

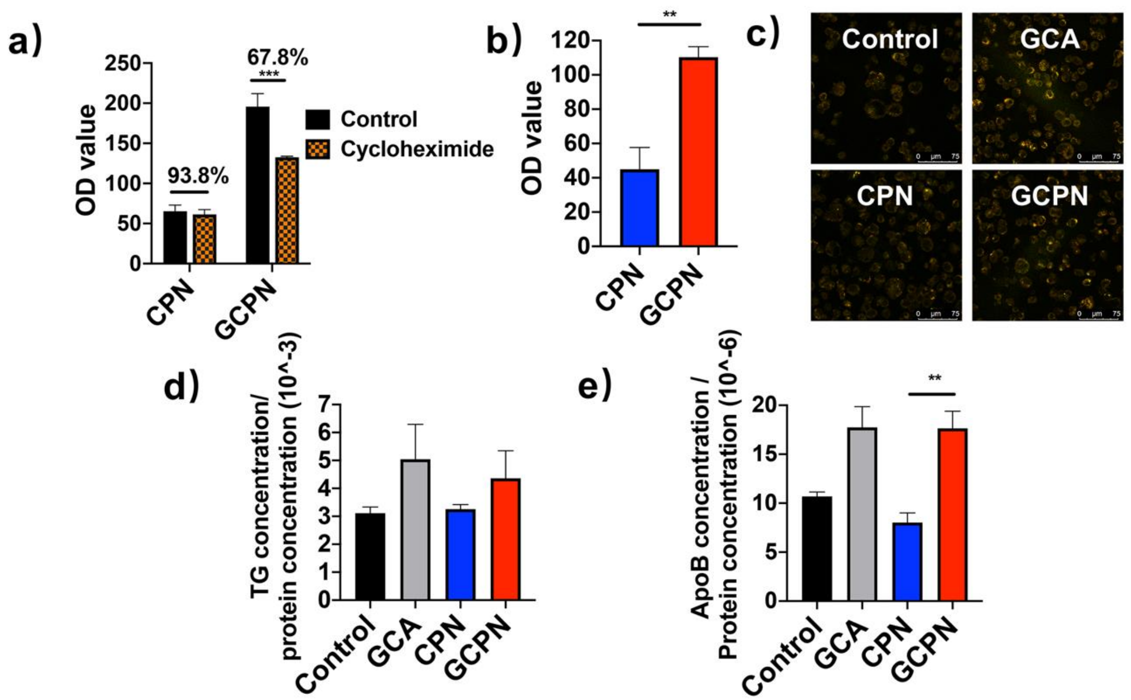

Figure S4. Testing on fat absorption pathway of CPN and GCPN in Caco-2 cells. a) Effect of cycloheximide on the transcytosis of CPN and GCPN. Cycloheximide did not block CPN transcytosis but reduced the transport of GCPN. b) Accumulation of CPN and GCPN in chylomicrons. c) CLSM images of chylomicron after treatment with lipids and GCA, CPN, or GCPN. GCA and GCPN increased the production of chylomicrons. d) Colorimetric analysis of TG secretion after treatment with lipids and GCA, CPN, or GCPN. GCA and GCPN increased the secretion of TG despite no significance due to the large SD value. e) ELISA study of the secretion of ApoB, the marker protein of TG after treatment with lipids and GCA, CPN, or GCPN. GCA and GCPN increased the secretion of ApoB. **, $p < 0.01$; ***, $p < 0.001$.

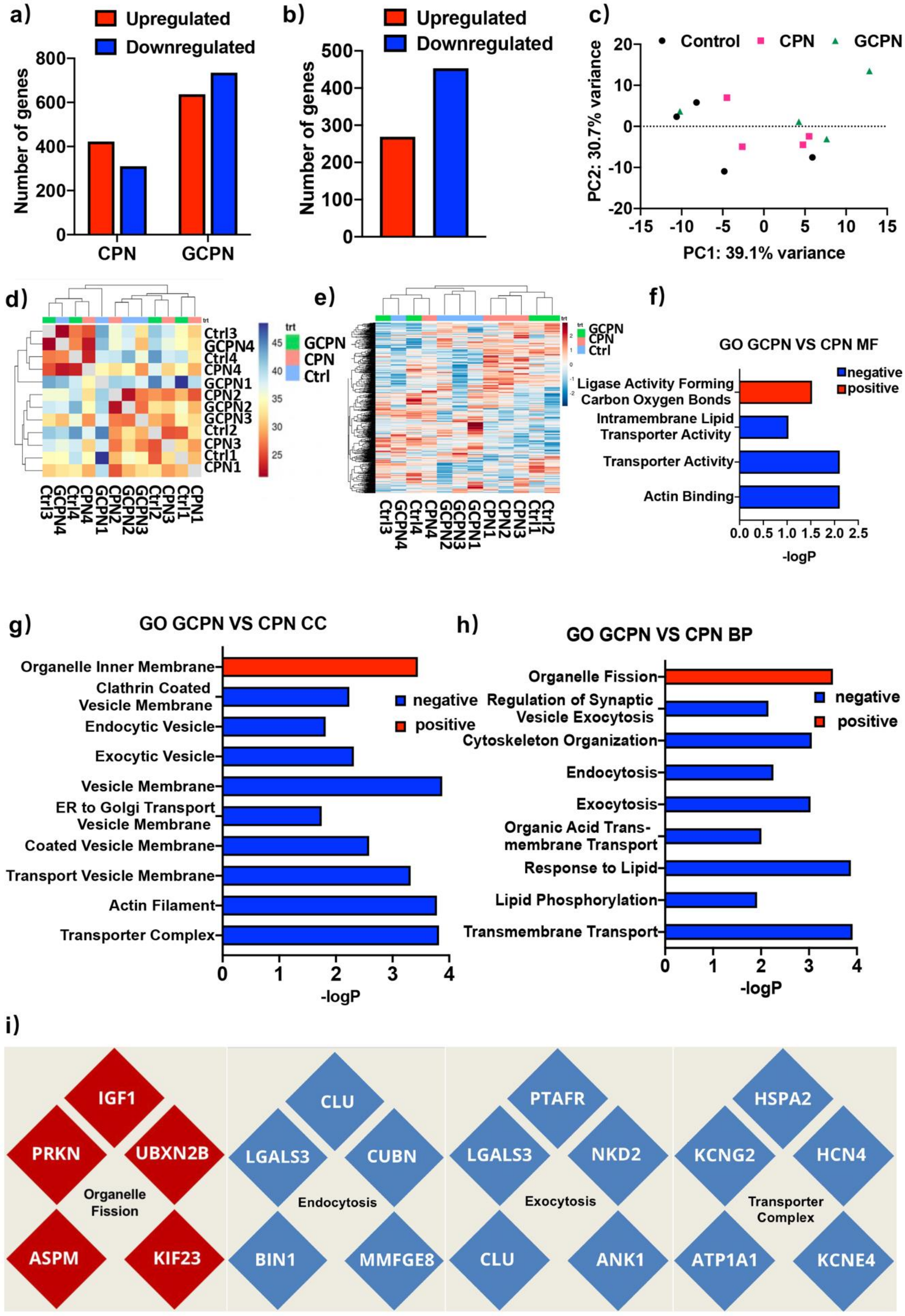

a)
Upregulated
Downregulated
Number of genes
800
600
400
200
0
CPN
GCPN
b)
Upregulated
Downregulated
Number of genes
500
400
300
200
100
0
c)
Control
CPN
GCPN
PC2: 30.7% variance
PC1: 39.1% variance
20
10
0
-10
-20
-15
-10
-5
0
5
10
15
d)
Ctrl3
GCPN4
Ctrl4
CPN4
GCPN1
CPN2
GCPN2
GCPN3
Ctrl2
CPN3
Ctrl1
CPN1
trt
GCPN
CPN
Ctrl
45
40
35
30
25
e)
trt
GCPN
CPN
Ctrl
Ctrl3
GCPN4
Ctrl4
CPN4
GCPN2
GCPN3
GCPN1
CPN1
CPN2
CPN3
Ctrl1
Ctrl2
f)
GO GCPN VS CPN MF
negative
positive
Ligase Activity Forming Carbon Oxygen Bonds
Intramembrane Lipid Transporter Activity
Transporter Activity
Actin Binding
0.0
0.5
1.0
1.5
2.0
2.5
-logP
g)
GO GCPN VS CPN CC
Organelle Inner Membrane
Clathrin Coated Vesicle Membrane
Endocytic Vesicle
Exocytic Vesicle
Vesicle Membrane
ER to Golgi Transport Vesicle Membrane
Coated Vesicle Membrane
Transport Vesicle Membrane
Actin Filament
Transporter Complex
negative
positive
0
1
2
3
4
-logP
h)
GO GCPN VS CPN BP
Organelle Fission
Regulation of Synaptic Vesicle Exocytosis
Cytoskeleton Organization
Endocytosis
Exocytosis
Organic Acid Trans-membrane Transport
Response to Lipid
Lipid Phosphorylation
Transmembrane Transport
negative
positive
0
1
2
3
4
-logP
i)
IGF1
PRKN
UBXN2B
Organelle Fission
ASPM
KIF23
CLU
LGALS3
CUBN
Endocytosis
BIN1
MMFGE8
PTAFR
LGALS3
NKD2
Exocytosis
CLU
ANK1
HSPA2
KCNG2
HCN4
Transporter Complex
ATP1A1
KCNE4

Figure S5. Genomics study of CPN and GCPN transport in Balb/c mice. a) Compared with control group, numbers of up- and downregulated genes after consecutive oral gavage of CPN and GCPN for two weeks. b) Compared with CPN group, number of up- and downregulated genes in GCPN group. c) PCA plot of sample distribution. d) Sample Euclidean distance among the control, CPN and GCPN. e) Heat map of the top 500 up- and downregulated genes in GCPN vs CPN. f-h) The statistical ORT based on the GO classification between GCPN vs CPN. The p-values of the listed genes classes were all <0.05. i) Top upregulated (red) and downregulated (blue) genes in the corresponding pathways.

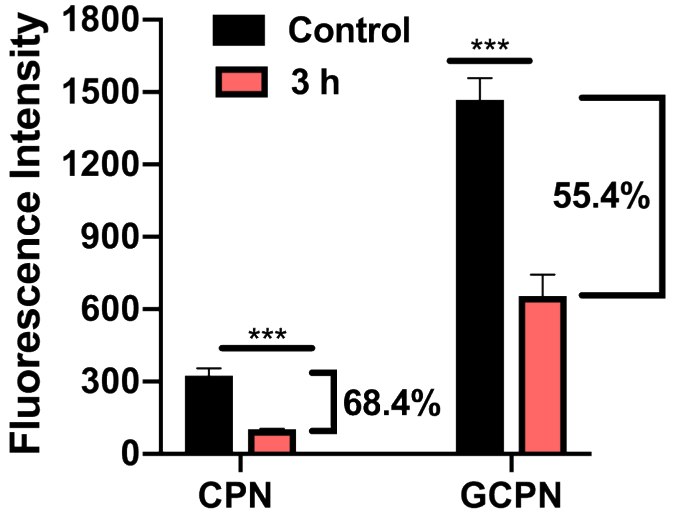

Figure S6. Exocytosis of CPN and GCPN in Caco-2 cells in 3 h.

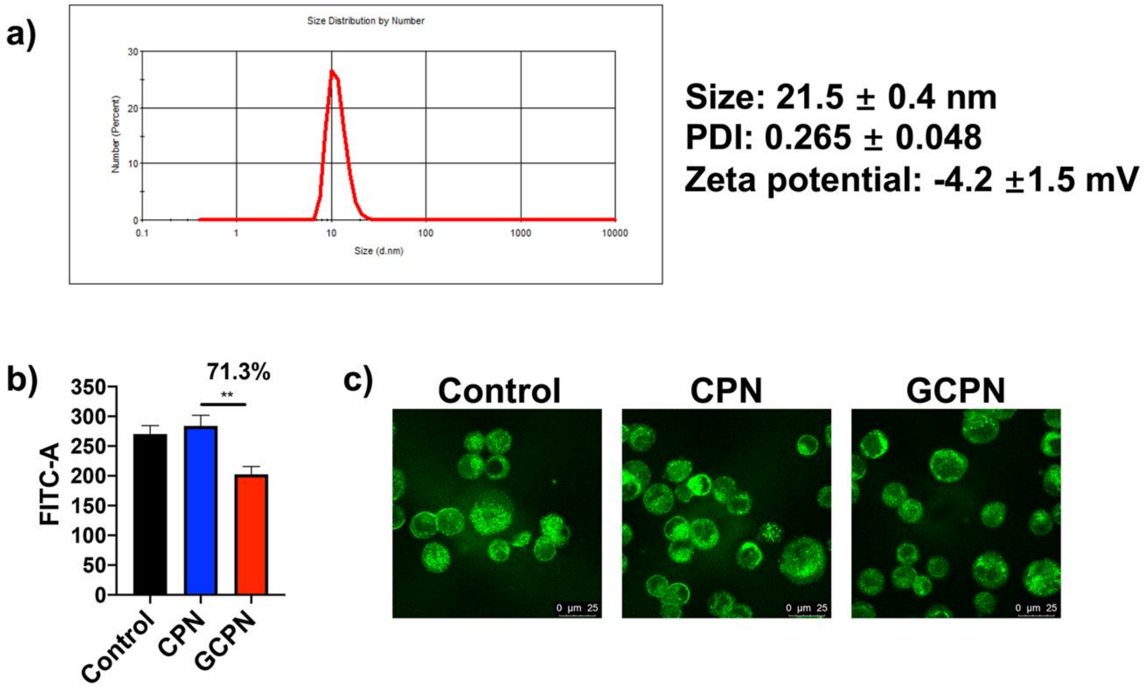

Figure S7. a) DLS analysis of C6-M; b) Flow cytometry and c) CLSM analysis of C6-M uptake in Caco-2 cells after CPN or GCPN incubation.

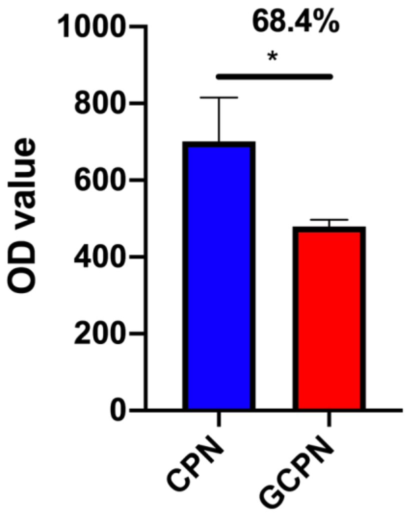

Figure S8. Flow cytometry analysis of B to A transcytosis of CPN and GCPN.

Video S1. 3D-Model of Caco-2 monolayers
Video S2. 3D-Model of CPN distribution through the Caco-2 monolayers
Video S3. 3D-Model of GCPN distribution through the Caco-2 monolayers
Video S4. ASBT distribution across the Caco-2 monolayer. ASBT (green) was mainly located in the apical side.

Table S1. The pharmacological inhibitors used in endocytosis pathway study.

Inhibitors	Concentration	Mechanisms
Chlorpromazine (CPZ)	30 μM	Blocking the assembly of subunit AP2 to the membrane, which is the prerequisite of recruitment of clathrin to form the clathrin-coated pit [1].
methyl-β-cyclodextrin (MβCD)	5 mM	Disrupting the lipid raft by cholesterol depletion while disturbing lipid raft-mediated endocytosis, including caveolae-mediated processes [2]
Nystatin	10 μg/mL	Inhibiting the caveolae-dependent endocytosis by disassembling caveolae and cholesterol in the membrane [3].
Genistein	100 μM	Inhibiting the caveolae-dependent endocytosis by interfering with the activity of tyrosine kinases [4].
5-(N-Ethyl-N-isopropyl)amiloride (EIPA)	20 μM	Inhibiting Na+/H+ exchanger and impeding the formation of macropinosomes, leading to the blocking of micropinocytosis [1].
Dynasore	80 μg/mL	A small GTPase inhibitor that blocks dynamin, the “molecular scissors” that assist the formation of vesicles from the plasma membrane [5-6].
Cytochalasin D (CytD)	0.5 μM	An actin filament inhibitor by depolymerizing the filament network, which is critical in scission and movement of vesicles away from the membrane by inducing phase separation of membrane lipid species and mechanical tension [7-9].

Table S2. Clathrin/Caveolae-independent endocytosis.

Routes	Description
Flotillin-1 (Flot-1)	A protein associated with lipid domains on cell membranes. It forms higher-order oligomers and recruits transmembrane proteins into the Flot-1 raft for endocytosis [10].
ADP-ribosylation factor (Arf6)	A small GTPase that can regulate actin polymerization and induce endocytosis via Arf6-enriched vesicles [11].
Ras homolog family member A (RhoA)	Belonging to Rho family GTPases and mainly assisting endocytosis [12].
Cell division control protein 42 homolog (Cdc42)	The regulator of the vesicle trafficking of various processes, including endocytosis, intracellular transport, and exocytosis [12].

Table S3. The oligonucleotide primer sequences in Real-Time PCR analysis.

Gene name	Sequence (5'-3')
Human β-actin	F: TCATCGGTATGGAGTCGGCG<br>R: CCGACATGACGTTGTTGGCA
Human Flot-1	F: CATTCTAACTCGCCTGCCAGA<br>R: CAAAGGCTTGTGATTCACCTGG
Human Arf6	F: AGGAGCTGCACCGCATTATC<br>R: CGTGGGGTTTCATGGCATCG
Human RhoA	F: ACACAGCTGGGCAGGAAGA<br>R: AAACTATCAGGGCTGTCGATGG
Human IBABP	F: AGG TTCTGAGAGCTGTGTTG<br>R: TTCTTCTCACTCTCCATCTCG

Table S4. The pharmacological inhibitors used in transcytosis pathway study.

Inhibitors	Concentration
Brefeldin	1 μM
Bafilomycin	0.5 μg/mL
17α-ethinylestradiol	50 μM

Table S5. The top 10 upregulated genes in CPN vs Control, GCPN vs Control and GCPN vs CPN after treatment in Caco-2 cells.

	Genes	Description
CPN vs Control	RNU4-1	RNA, U4 small nuclear 1
	MT-TL1	mitochondrially encoded tRNA-Leu (UUA/G) 1
	ZMAT5	zinc finger matrin-type 5
	AC092828.1	novel transcript
	IFIT1	interferon induced protein with tetratricopeptide repeats 1
	CBR3-AS1	CBR3 antisense RNA 1
	MT-ND6	mitochondrially encoded NADH:ubiquinone oxidoreductase core subunit 6
	GTF2IP20	general transcription factor IIi pseudogene 20
	TLCD1	TLC domain containing 1
	RPS15P4	ribosomal protein S15 pseudogene 4
GCPN vs Control	MX1	MX dynamin like GTPase 1
	MT-ND6	mitochondrially encoded NADH:ubiquinone oxidoreductase core subunit 6
	OAS3	2'-5'-oligoadenylate synthetase 3
	LINC00342	long intergenic non-protein coding RNA 342
	ANKRD36C	ankyrin repeat domain 36C
	MT-ND2	mitochondrially encoded NADH:ubiquinone oxidoreductase core subunit 2
	MT-ND5	mitochondrially encoded NADH:ubiquinone oxidoreductase core subunit 5
	RPL36A	ribosomal protein L36a
	ANKRD20A11P	ankyrin repeat domain 20 family member A11, pseudogene
	MT-ATP8	mitochondrially encoded ATP synthase membrane subunit 8
GCPN vs CPN	MT-ND2	mitochondrially encoded NADH:ubiquinone oxidoreductase core subunit 2
	MT-ATP8	mitochondrially encoded ATP synthase membrane subunit 8
	PDK1	pyruvate dehydrogenase kinase 1
	PDCD5	programmed cell death 5
	MT-ND5	mitochondrially encoded NADH:ubiquinone oxidoreductase core subunit 5
	MT-ATP6	mitochondrially encoded ATP synthase membrane subunit 6
	RPL36A	ribosomal protein L36a
	MT-ND4	mitochondrially encoded NADH:ubiquinone oxidoreductase core subunit 4
	CCDC34	coiled-coil domain containing 34
	GTF2IP13	general transcription factor IIi pseudogene 13

Table S6. The top 10 upregulated genes in CPN vs Control, GCPN vs Control and GCPN vs CPN after treatment in ileum of Balb/c mice.

	Genes	Description
CPN vs Control	PPM1J	protein phosphatase 1J
	IFIT1BL2	interferon induced protein with tetratricopeptide repeats 1B like 2
	PDP2	pyruvate dehyrogenase phosphatase catalytic subunit 2
	GNG7	guanine nucleotide binding protein (G protein), gamma 7
	FNIP1	folliculin interacting protein 1
	GM10800	predicted gene 10800
	LRRN4CL	LRRN4 C-terminal like
	UNC93A	unc-93 homolog A
	IRS2	insulin receptor substrate 2
	PPARGC1A	peroxisome proliferative activated receptor, gamma, coactivator 1 alpha
GCPN vs Control	NR1D1	nuclear receptor subfamily 1, group D, member 1
	STOM	stomatin
	CD36	CD36 molecule
	ANGPTL8	angiopoietin-like 8
	UNC93A	unc-93 homolog A
	PPARG	peroxisome proliferator activated receptor gamma
	FZD4	frizzled class receptor 4
	LRRN4CL	LRRN4 C-terminal like
	ME1	malic enzyme 1, NADP(+)-dependent, cytosolic
	DNASE1	deoxyribonuclease I
GCPN vs CPN	ANGPTL8	angiopoietin-like 8
	DUSP4	dual specificity phosphatase 4
	NR1D1	nuclear receptor subfamily 1, group D, member 1
	1700119H24RIK	RIKEN cDNA 1700119H24 gene
	RAD9B	RAD9 checkpoint clamp component B
	PPARG	peroxisome proliferator activated receptor gamma
	TLCD1	TLC domain containing 1
	GLIPR2	GLI pathogenesis-related 2
	CLDN8	claudin 8
	MFAP5	microfibrillar associated protein 5

References

[1] N. Wytinck, D. S. Sullivan, K. T. Biggar, L. Crisostomo, P. Pelka, M. F. Belmonte, S. Whyard, *Sci Rep-Uk* **2020**, *10* (1).

[2] N. E. Rodriguez, U. Gaur, M. E. Wilson, *Am J Trop Med Hyg* **2005**, *73* (6), 177.

[3] X. D. Zhu, Y. Zhuang, J. J. Ben, L. L. Qian, H. P. Huang, H. Bai, J. H. Sha, Z. G. He, Q. Chen, *J Biol Chem* **2011**, *286* (10), 8231.

[4] F. Chen, L. Y. Zhu, Y. L. Zhang, D. Kumar, G. L. Cao, X. L. Hu, Z. Liang, S. L. Kuang, R. Y. Xue, C. L. Gong, *Sci Rep-Uk* **2018**, *8*.

[5] S. M. Ferguson, P. De Camilli, *Nat Rev Mol Cell Bio* **2012**, *13* (2), 75.

[6] E. Macia, M. Ehrlich, R. Massol, E. Boucrot, C. Brunner, T. Kirchhausen, *Dev Cell* **2006**, *10* (6), 839.

[7] O. L. Mooren, B. J. Galletta, J. A. Cooper, *Annu Rev Biochem* **2012**, *81*, 661.

[8] N. Ben-Dov, R. Korenstein, *Exp Cell Res* **2013**, *319* (7), 946.

[9] C. Yu, K. Achazi, L. Moller, J. D. Schulzke, M. Niedrig, R. Bucker, *Plos One* **2014**, *9* (5).

[10] M. Meister, R. Tikkanen, *Membranes (Basel)* **2014**, *4* (3), 356.

[11] T. Van Acker, J. Tavernier, F. Peelman, *Int J Mol Sci* **2019**, *20* (9).

[12] X. Chi, S. Wang, Y. Huang, M. Stamnes, J. L. Chen, *Int J Mol Sci* **2013**, *14* (4), 7089.
